# Cannabidiol directly targets mitochondria and disturbs calcium homeostasis in acute lymphoblastic leukemia

**DOI:** 10.1038/s41419-019-2024-0

**Published:** 2019-10-14

**Authors:** Miguel Olivas-Aguirre, Liliana Torres-López, Juan Salvador Valle-Reyes, Arturo Hernández-Cruz, Igor Pottosin, Oxana Dobrovinskaya

**Affiliations:** 1 Laboratory of Immunobiology and Ionic Transport Regulation, University Center for Biomedical Research, University of Colima, Av. 25 de Julio 965, Col. 28030 Colima, Mexico; 2 Department of Cognitive Neuroscience and National Laboratory of Channelopathies (LaNCa), Institute of Cellular Physiology, National Autonomous University of Mexico, Mexico-City, Mexico

**Keywords:** Drug development, Cell death, Calcium signalling, Target identification, Acute lymphocytic leukaemia

## Abstract

Anticancer properties of non-psychoactive cannabinoid cannabidiol (CBD) have been demonstrated on tumors of different histogenesis. Different molecular targets for CBD were proposed, including cannabinoid receptors and some plasma membrane ion channels. Here we have shown that cell lines derived from acute lymphoblastic leukemia of T lineage (T-ALL), but not resting healthy T cells, are highly sensitive to CBD treatment. CBD effect does not depend on cannabinoid receptors or plasma membrane Ca^2+^-permeable channels. Instead, CBD directly targets mitochondria and alters their capacity to handle Ca^2+^. At lethal concentrations, CBD causes mitochondrial Ca^2+^ overload, stable mitochondrial transition pore formation and cell death. Our results suggest that CBD is an attractive candidate to be included into chemotherapeutic protocols for T-ALL treatment.

## Introduction

Acute lymphoblastic leukemia (ALL) of T lineage (T-ALL) represents a highly aggressive cancer, resistant to chemotherapy and with increased risk of relapse, which occurs in 15% of childhood and 25% of adult ALL cases. In T-ALL, a long-term remission fails in ~20% of children and 40% of adult patients. The prognosis for these groups remains poor, with a 5-year survival rate of <25%^1–5^. Thus, novel therapeutic strategies for the T-ALL treatment are needed.

Cannabinoids are a group of more than 60 structurally related terpenophenolic compounds. Most of them exert their effects through cannabinoid CB1 and CB2 receptors, which belong to the family of G protein-coupled receptors. Lymphocytes mainly express CB2, whereas CB1 are highly expressed in the central nervous system (CNS). Cannabinoids have been used for decades in the field of palliative care. Recently two phytocannabinoids, Δ^9^-tetrahydrocannabinol (THC) and cannabidiol (CBD), were found to possess antitumorigenic properties^6,7^. Contrary to THC, a well-known strong CB1 agonist with psychotropic effect, CBD has a low binding affinity for CB1/2 receptors and is considered as a “non-intoxicating drug”^8^. Molecular targets for CBD are uncertain. Among putative candidates, some members of the TRP channels´ family, orphan cannabinoid receptor GPR55, 5HT and PPARγ receptors have been proposed^6,9^.

In the present study we addressed the immediate targets for CBD in leukemic cell lines, derived from T-ALL patients in relapse.

## Results

### CBD suppresses viability and impairs migration of leukemic T cells

**Fig. 1:**
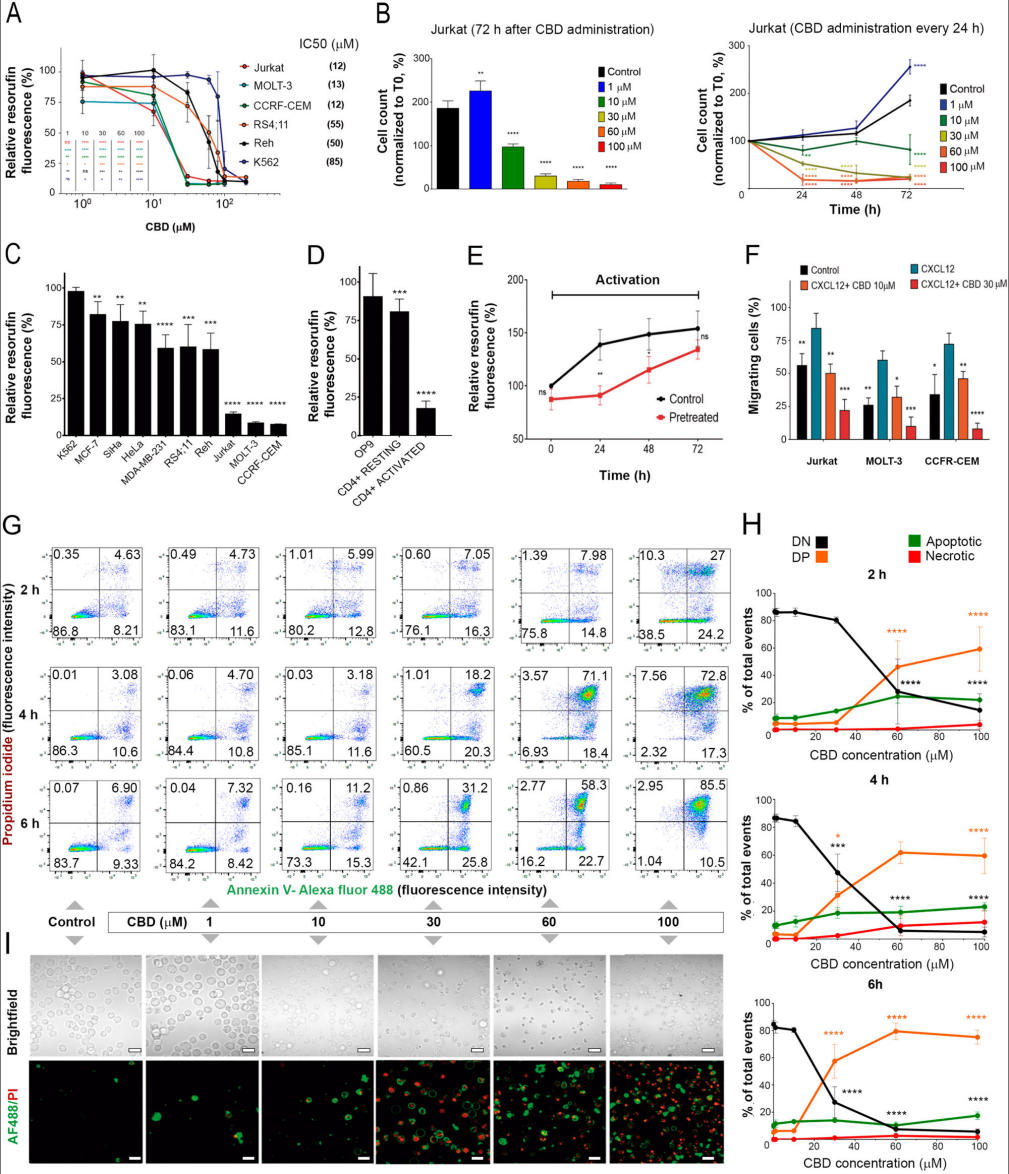
CBD effect on viability in different cancer cell lines. **a** Cell viability, evaluated by resazurin-based metabolic assay, as a function of CBD concentration in human leukemic cell lines of different lineages at 24 h of treatment. Cell lines derived from T-ALL (Jurkat, MOLT-3, and CEM), B-ALL (RS4;11 and Reh) and CML (K562) were tested. Data (resorufin fluorescence intensity, arbitrary units) were normalized to the vehicle-treated control and shown as mean ± SD (*n* = 8; *p < 0.05; **p < 0.01; ***p < 0.001; ****p < 0.0001; ns, not significant; one-way ANOVA). **b** Live cell count (trypan blue exclusion test) in long-lasting Jurkat cells cultures exposed to different CBD concentrations (0–100 μM). Left: cell count at 72 h after single CBD administration; right: fresh CBD was added, 50% of medium volume was changed and cells were counted every 24 h. Data are normalized to the initial point (0 h) and shown as mean ± SD (*n* = 3; **p < 0.01; ***p < 0.001; ****p < 0.0001, one-way ANOVA test). **c** Cell viability was evaluated by resazurin-based metabolic assay at 24 h of treatment with CBD (30 μM) in human tumor cell lines of different histogenesis, including CML (K562), B-ALL (Reh and RS4;11), T-ALL (CEM, MOLT-3, and Jurkat), cervical cancer (SiHa and HeLa), and breast cancer (MCF7-7 and MDA-MB-231). Data (resorufin fluorescence intensity) were normalized to vehicle-treated control and reported as mean ± SD (*n* = 8 of four independent experiments; (*p < 0.05; **p < 0.01; ***p < 0.001; ****p < 0.0001, Student’s *t*-test). **d** Cell viability was evaluated by resazurin-based metabolic assay at 24 h of treatment with CBD (30 μM) in non-cancerous cells. Human CD4^+^ cells were activated with anti-CD3/CD28 antibodies as described in Materials and Methods section. Data (resorufin fluorescence intensity) are normalized to the vehicle-treated control and reported as mean ± SD (*n* = 8 in at least three independent experiments; *p < 0.05; **p < 0.01; ***p < 0.001; ****p < 0.0001; Student’s *t*-test). **e** After preincubation with CBD (30 μM, 24 h), non-cancerous CD4^+^ cells were activated by anti-CD3/antiCD28 antibodies. Cell viability was evaluated by resazurin-based metabolic assay every 24 h. Data (resorufin fluorescence intensity) were normalized to 0 h time point and shown as mean ± SD (*n* = 8 in at least three independent experiments). Statistical comparison between control and pretreated samples was undertaken at each time point (*p < 0.05; **p < 0.01; ***p < 0.001; ****p < 0.0001, Student’s *t*-test). **f** Migration capacity of Jurkat cells pretreated with CBD (10 or 30 μM, 2 h) was evaluated by chemotactic migration assay, using a Transwell system. Cells were allowed to migrate for 4 h, CXCL12 was used as a chemoattractant. The percentage of migrated cells was determined by cells count in the lower chamber. Data are mean ± SD (*n* = 4). Statistical comparison was made with respect to positive control (CXCL12) (*p < 0.05; **p < 0.01; ***p < 0.001; ****p < 0.0001, one-way ANOVA test). **g**–**i** Cell death was evaluation by flow cytometry (**g**, **h**) and fluorescent microscopy (**I**) in Jurkat cells treated with different concentrations of CBD using Annexin V-AF488/PI double staining. Representative dot plots (2, 4, and 6 h) and images (12 h) are shown in **g** and **i**, correspondingly. Scale bar: 20 μm. In every dot plot lower left quadrant represents Annexin V^−^PI^−^ (DN) live cells, in the lower right quadrant are Annexin V^+^PI^−^ (early apoptotic) cells, Annexin V^−^PI^+^ (primary necrotic) cells are in the upper left quadrant, whereas the double-stained population Annexin V^+^PI^+^ (DP) in the upper right quadrant represents dead cells, which may include necrotic and late apoptotic ones. Statistical analysis of flow cytometry data is given in **h** (*n* = 3; *p < 0.05; **p < 0.01; ***p < 0.001; ****p < 0.0001, one-way ANOVA test)

Cytotoxic effect of CBD was first evaluated in leukemic cell lines of different lineages, including T-ALL, B lineage-derived ALL (B-ALL), and chronic myelogenous leukemia (CML) by means of metabolic assay. T-ALL were the most CBD-sensitive among tested cell lines (Fig. 1a). However, there is an uncertainty in the interpretation of this result, which may be explained by mitochondrial metabolism inhibition, decreased proliferation rate, increased level of cell death, or combination of these effects. Then survival and proliferation of CBD- treated Jurkat cells were evaluated in long-lasting (72 h) cultures by counting live and dead cells CBD at 30–100 μM induced cell death, while at 10 μM the cells remained alive, but did not proliferate. Noteworthy, at low (1 μM) concentration, CBD stimulated the cell proliferation (Fig. 1b, left). Stimulation was also obtained in experiments with a sequential (1 μM every 24 h) CBD administration (Fig. 1b, right). Strikingly, dose dependence was very similar in both cases, irrespective to supposed CBD accumulation during sequential application. Apparently, cell fate was defined already by the first CBD administration.

In additional experiments, cervical and breast cancer cell lines were included for a comparison, because CBD effect on these types of cancer was reported earlier. Again, among 10 cell lines tested, cytotoxic effect of CBD was the most pronounced in T-ALL (Fig. 1c).

We also tested CBD toxicity in non-tumoral cells. Murine bone marrow-derived cell line OP9 displayed high resistance to CBD (Fig. 1d). Strikingly, in contrast to activated human CD4^+^ T cells, resting CD4^+^ T cells were relatively insensitive to CBD. Noteworthy, resting CD4^+^ T cells pretreated with CBD (30 μM, 24 h) were able to respond to activating stimuli. At 72 h of activation their proliferative response was statistically indistinguishable from that of untreated CD4^+^ cells (Fig. 1e).

The infiltration of lymph nodes and CNS by leukemic cells and formation of a mediastinal mass are tightly related to disease relapse and unfavorable prognosis in T-ALL^10^. Thus, the effect of CBD on the T-ALL cells migration was addressed. Since CXCL12/CXCR4 axis plays a significant role in the dissemination of leukemic cells^11,12^, CXCL12 was used as a chemoattractant. Migration of T-ALL cells was shown to be significantly suppressed by 2 h preincubation with 30 μM CBD (Fig. 1f).

CBD (30–100 μM) triggered apoptosis in a part of the cell population (Annexin V - single positive cells). At the same time, loss of plasma membrane integrity was observed in a large cell population as early as at 4 h of treatment (Annexin V/ propidium iodide (PI) – double positive cells), indicating necrosis (Fig. 1g–i).

**Fig. 2:**
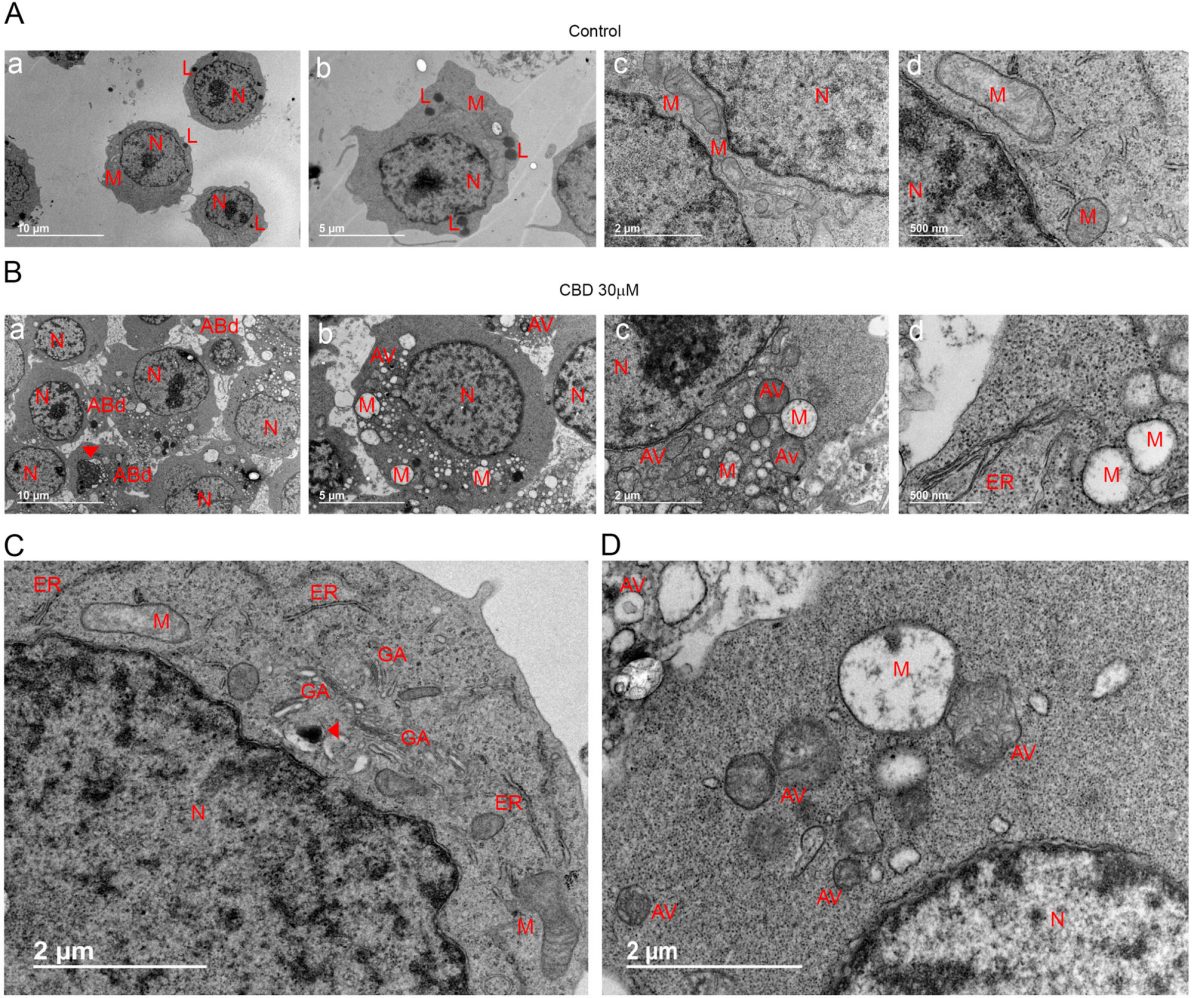
CBD induces ultrastructural alterations in Jurkat cells. **a**, **b** Representative TEM images of untreated Jurkat cells (**a**) and Jurkat cells, fixed after 2 h of incubation with CBD (30 μM, **b**). Scale bars are indicated. **c**, **d** High-resolution TEM images of control (**c**) and CBD-treated cells (**d**). Scale bar: 2 μm. Cell components are indicated as following: nucleus (N), Golgi apparatus (GA), mitochondria (M), endoplasmic reticulum (ER), and autophagic vacuoles (AV). Note autophagic bodies (ABd) in **b**, panel a; inclusion of condensed chromatin in one of ABds is indicated by arrow. An electron dense lysosome in the neighborhood of an autophagic vacuole is indicated by arrow in **c**

Transmission electron microscopy (TEM) imaging revealed multiple dramatic changes in cell morphology already after 2 h of CBD (30 μM) exposure (Fig. 2). Two of the most characteristic features were extensive cytoplasmic vacuolation and numerous swollen mitochondria, devoid of cristae. Golgi complex and endoplasmic reticulum (ER) were disassembled, which seems to be related to cytoplasmic vacuolation. Plasma membrane blebbing and apoptotic bodies (ApoBDs) formation were detected. The observed morphological changes pointed out to a complex mechanism, with both apoptotic and necrotic symptoms. In addition, double membrane vacuoles, containing degrading material (autophagosomes), were much more numerous in CBD-treated cells as compared to control.

### Sublethal CBD concentrations activate autophagy in leukemic cells

**Fig. 3:**
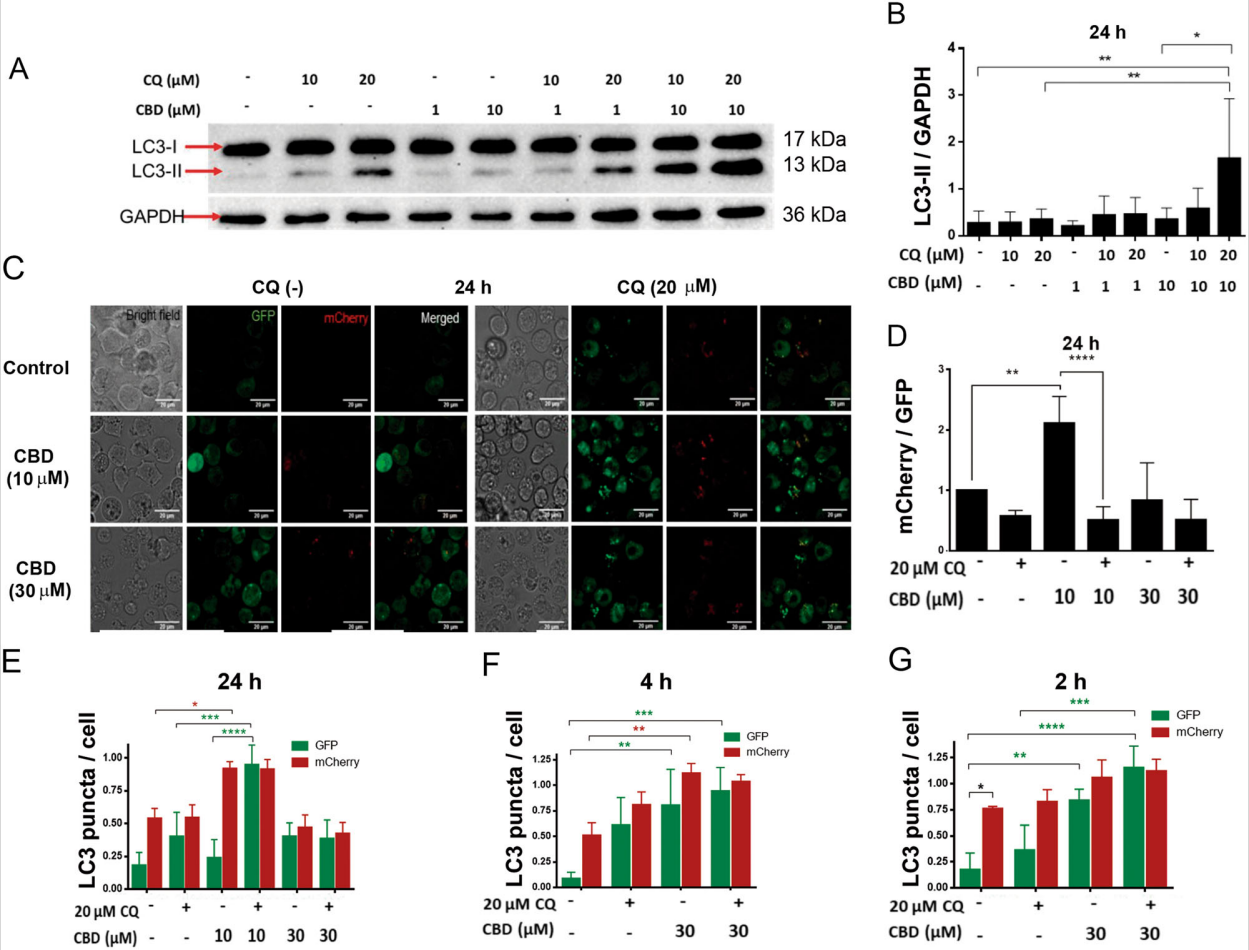
CBD induces autophagy in Jurkat cells. **a** Representative Western blot of LC3-I/II protein expression in vehicle- or CBD- treated Jurkat cells (24 h). CQ was used to prevent the fusion of autophagosomes with lysosomes, where LC3-II is otherwise degraded. The concentrations of CQ and CBD are indicated. GAPDH was used as a loading control. Note that in absence of CQ the accumulation of LC3-II was observed neither in control (basal autophagy) nor in CBD-treated samples. In the presence of CQ LC3-II is accumulated and corresponding bands are more intense. **b** LC3-II/GAPDH density ratio in samples, obtained from Jurkat cells, grown in different conditions (as in example shown in **a**). Data are mean ± SD (*n* ≥ 4; *p < 0.05; **p < 0.01). Control and different treatments were compared, using one-way ANOVA with Tukey’s multiple comparisons tests. **c** Confocal microscopy images of mCherry-GFP Jurkat cells, incubated over 24 h at different conditions as indicated. CQ was used to prevent the fusion of autophagosomes with lysosomes during the autophagy flux. Note that the inhibition of autophagolysosomes formation by CQ results in prevention of GFP quenching in the acidic environment within a lysosome. **d** mCherry/GFP puncta ratio was calculated from confocal images of Jurkat cells as shown in **c**, incubated at different conditions. Quantification of the average number of mCherry and GFP puncta was performed using ImageJ “Particle analysis” tool. For each condition, 3 fields (10–20 cells/ field) were analyzed. Data from three independent experiments were averaged and are shown as mean ± SD. One-way ANOVA with Tukey’s post-hoc tests were employed to compare statistical significance between groups (**p < 0.01; ****p < 0.0001). **e**–**g** LC3 puncta counts, where GFP and mCherry puncta were counted and analyzed separately in Jurkat cells in control samples or in samples treated with different combinations of CBD and CQ, and incubated during 24 h (**e**), 4 h (**f**) or 2 h (**g**). Quantification of the average number of mCherry and GFP puncta was performed using ImageJ “Particle analysis” tool. For each condition, 3 fields (10–20 cells/field) were analyzed. Data from four independent experiments were averaged and are shown as mean ± SD. Significance is depicted in green, when comparing GFP puncta between groups and in red for mCherry puncta comparison. Significance in black represents differences between GFP/mCherry puncta (*n* ≥ 4, *p < 0.05; **p < 0.01; ***p < 0.001; ****p < 0.0001; two-way ANOVA with Tukey’s post-hoc tests)

Autophagy is a catabolic cellular process, representing an important strategy to ensure cell homeostasis by the elimination of defunct organelles in both physiological and pathological conditions^13^. Basal levels of autophagy serve a housekeeping function, whereas stress stimulates autophagy. CBD was shown to cause cell death by inducing a crosstalk between apoptosis and autophagy in breast cancer^14^. To reveal the ability of CBD to induce autophagy in T-ALL, LC3-I to LC3-II conversion and LC3-II turnover were monitored by immunoblotting. In the course of autophagy, microtubule-associated protein LC3-I first conjugates with phosphatidylethanolamine, forming LC3-II, followed by the LC3-II translocation to autophagosomes. An increased LC3-II level is considered as an indicator of the autophagy, but LC3-II is degraded by lysosomal proteases after autophagosome-lysosome fusion. Chloroquine (CQ) is known to prevent autophagosome-lysosome fusion and to inhibit the LC3-II degradation, thus LC3-II is accumulated in the presence of CQ^15,16^. Western blot analysis of LC3 variants demonstrated that sublethal CBD concentration (10 µM) effectively induced autophagy in Jurkat cells (Fig. 3a, b), since LC3-II level was higher in cells treated with both CBD (10 µM) and CQ (20 µM) when compared to either 20 µM CQ (basal autophagy) or 10 µM CBD treatment alone. Note that accumulation of LC3-II was diminished by its degradation during the autophagic flux in the samples without CQ.

Proceeding of the autophagic flux at the single-cell level was monitored using mCherry-GFP-LC3 expressing Jurkat cells. The mCherry-GFP-LC3 experimental strategy is based on the fact that GFP is quenched at acidic pH, found in the autophagolysosome, while mCherry is not. Accordingly, enhancement of the mCherry/GFP ratio sensitive to CQ indicates the autophagolysosome formation^17^. Figure 3c shows representative images from the experiment, in which Jurkat GFP-mCherry-LC3 cells were incubated with CBD (10 and 30 µM) and CQ, alone or in combination. High mCherry/GFP ratio, efficiently equalized by CQ, was observed in samples, treated with 10 µM CBD (Fig. 3d, e), evidencing a throughput of the autophagic pathway. In samples treated with 30 µM CBD this ratio was low (Fig. 3d, e). However, the number of autophagosomes was increased at early times after CBD administration (30 µM), as it was revealed by an enhanced level of both GFP^+^ and mCherry^+^ puncta (Fig. 3f, g). These data may be explained by a truncation of basal, and, possibly, also CBD-induced autophagy at late phases in damaged cells.

### CBD causes mitochondrial damage and induces cytochrome C release

Dissipation of the mitochondrial transmembrane potential (ΔΨm) is a hallmark of the mitochondrial permeability transition (MPT) - driven necrosis as well as of intrinsic apoptosis. It involves an irreversible mitochondrial outer membrane permeabilization and a release of various pro-apoptotic factors, including cytochrome C (Cyt-C), to the cytosol. In the cytosol, Cyt-C contributes to the apoptosome formation, with a consequent activation of the initiator caspase 9, which cleaves and activates executioner caspases^18^.

**Fig. 4:**
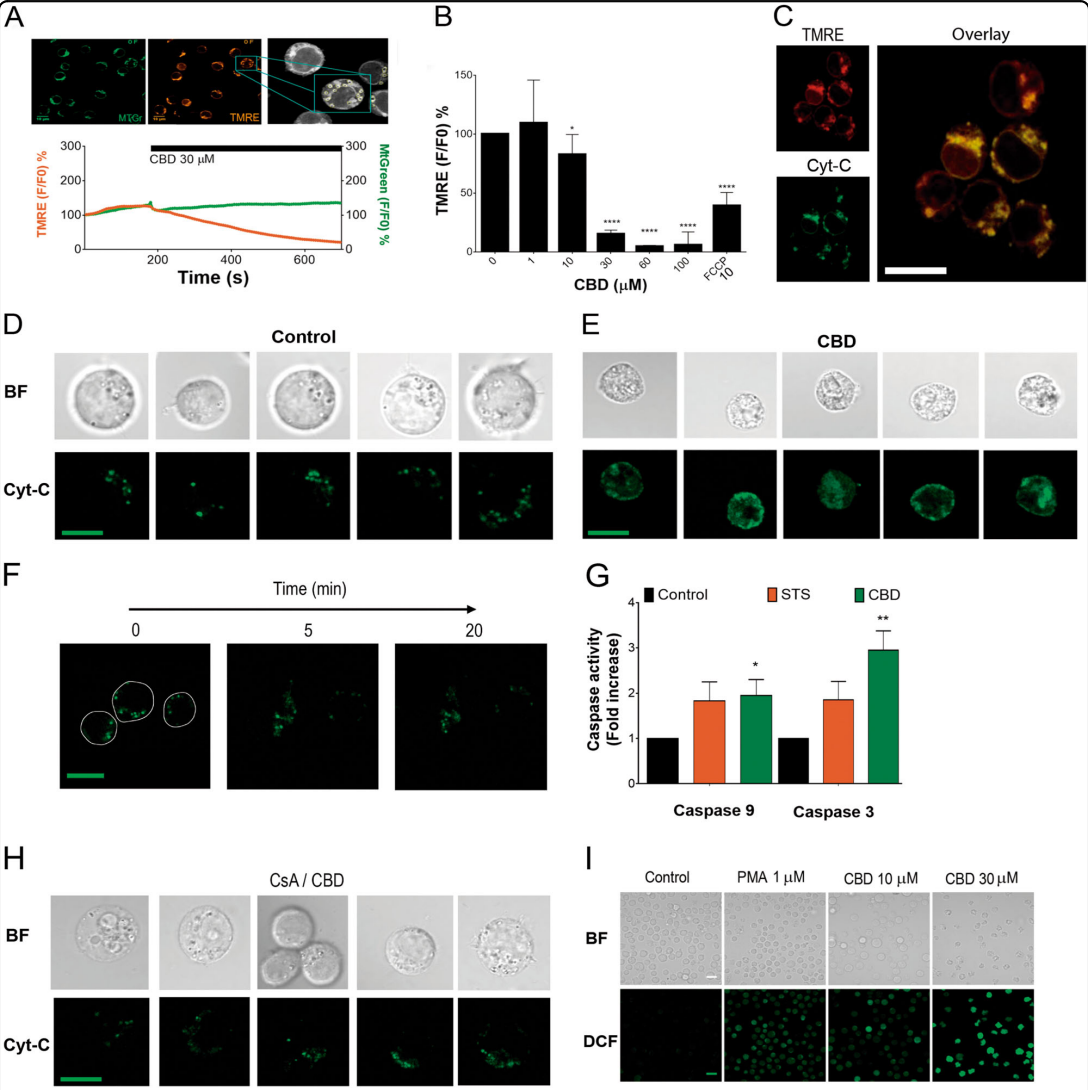
CBD impairs mitochondrial function, induces Cyt-C liberation and ROS production. **a** Monitoring of Δψm loss in CBD-treated leukemic cells. Jurkat cells were double-stained with MtGreen and TMRE, mitochondria (ROI) were selected (upper panel) and intensity of fluorescence was monitored by confocal microscopy after CBD (30 μM) administration (lower panel, *n* = 36; see also Supplementary movie 1). **b** Intensity of TMRE fluorescence as an indicator of Δψm in Jurkat cells, treated with different concentrations of CBD (0–100 μM) during 10 min. FCCP (10 μM) was used as a positive control. Data are mean ± SD (*n* = 8 in three independent experiments). Statistical comparison with control was performed by means of one-way ANOVA test (*p < 0.05; ****p < 0.0001). **c** EYFP-Cyt-C is localized in mitochondria of Jurkat cells after 12 h of transfection. EYFP-Cyt-C and TMRE fluorescence are colocalized (200 nM TMRE, 200 ng EYFP-Cyt-C, pseudocolor). Images were acquired by confocal microscopy at 12 h after transfection, scale bar: 10 μm. **d** Representative images of five Jurkat cells, transfected with EYFP-Cyt-C (as in **c**). Dense green fluorescent puncta (pseudocolor) reflect Cyt-C localization in intact mitochondria. Scale bar: 10 μm. **e** Representative images of five Jurkat cells, transfected with EYFP-Cyt-C (as in **c**) and treated with CBD (30 μM, 1 h). Cyt-C was released from mitochondria as evidenced by a diffuse EYFP-CytC distribution. Scale bar: 10 μm. **f** EYFP-Cyt-C distribution in Jurkat cells, expressing EYFP-Cyt-C, at 0, 5, and 20 min with CBD (30 μM). Scale bar: 10 μm. **g** Caspase-9 and caspase-3 activity in vehicle- and CBD- treated (30 μM, 12 h) Jurkat cells. Staurosporine (STS, 5 μM) was used as a positive control. After incubation, cells were lysed, and caspase activity was determined by colorimetric assay (BioVision). Fold increase in activity compared to control was plotted as mean ± SE (four independent experiments). One-way ANOVA test was performed to compare CBD-treated to control group (*p < 0.05; **p < 0.01). **h** Pretreatment with mPTP inhibitor CsA (10 μM) prevents Cyt-C release, induced by CBD (30 μM; 1 h). Compare these images with Fig. 4d, e (vehicle- and CBD-treated cells) and note the protective effect of CsA. Scale bar: 10 μm. **i** ROS production as evaluated by fluorescent microscopy in DCF-loaded (2 μM) Jurkat cells. CBD effect on ROS production was monitored after 1 h of incubation. PMA (1 μM) was used as a positive control

We monitored ΔΨm in Jurkat cells double-stained with green fluorescent dye MtGreen, which covalently binds to mitochondrial matrix proteins, and tetramethylrhodamine ethyl ester (TMRE), a cationic fluorescent dye that is readily sequestered by energized mitochondria (Fig. 4a). When exposed to CBD (30 μM), the intensity of TMRE fluorescence, in contrast to that of MtGreen, was gradually decreased within the first 10 min of treatment, indicating a rapid ΔΨm loss (Fig. 4a, Supplementary Movie 1). The loss of TMRE fluorescence was dose-dependent (Fig. 4b). To monitor Cyt-C release from mitochondria in a response to CBD treatment, Jurkat cells were transfected with EYFP-Cyt-C. In untreated cells, Cyt-C localization was restricted to mitochondria, as confirmed by punctate distribution of EYFP-Cyt-C staining and its colocalization with TMRE (Fig. 4c, d). After treatment with CBD (30 μM), EYFP-Cyt-C distribution in the cytoplasm of treated cells became more diffuse (Fig. 4e), indicating Cyt-C release from mitochondria, observed as early as within the first 20 min of treatment (Fig. 4f). Concomitantly, cell volume was significantly reduced, as it was previously reported for CBD-treated Jurkat cells by others^19^. Jurkat cells, exposed to CBD, exhibited an enhanced activity of caspases 9 and 3, confirming the triggering of intrinsic apoptotic pathway (Fig. 4g).

Collapse of the ΔΨm is frequently associated with the induction of the mitochondrial permeability transition pore (mPTP), a wide channel formed through inner and outer mitochondrial membranes. The formation of mPTP is potently blocked by cyclosporine A (CsA)^20^. Two distinct mechanisms, leading to the Cyt-C release from mitochondria were suggested: one is related to the mPTP and inhibited by CsA and another is Bax-dependent but CsA-insensitive^21,22^. In our experiments both CBD-induced phenomena, Cyt-C release from mitochondria and cell shrinkage were inhibited by CsA (Fig. 4h). mPTP opening is associated with the oxidative stress^20^. CBD-induced reactive oxygen species (ROS) overproduction was previously reported in different cell models^14,23^. As shown in the Fig. 4i, CBD within few minutes provoked a dose-dependent increase in ROS generation.

### CBD-induced mitochondrial Ca^2+^ overload is responsible for the formation of mPTP

CBD-mediated elevation of the cytosolic free Ca^2+^ ([Ca^2+^]_i_) has been observed in several cancer and non-cancerous cells^24,25^. Calcium signal signature defines the cell fate, survival, or death scenarios^26^. Elevated intramitochondrial Ca^2+^ ([Ca^2+^]_m_) is a prerequisite for the mPTP formation^20^. It is generally assumed that [Ca^2+^]_m_ increase is triggered by an increase of [Ca^2+^]_i_. In many cases, Ca^2+^ source can be ER, whose membranes come to a very close proximity with the outer mitochondrial membrane (OMM)^27^.

**Fig. 5:**
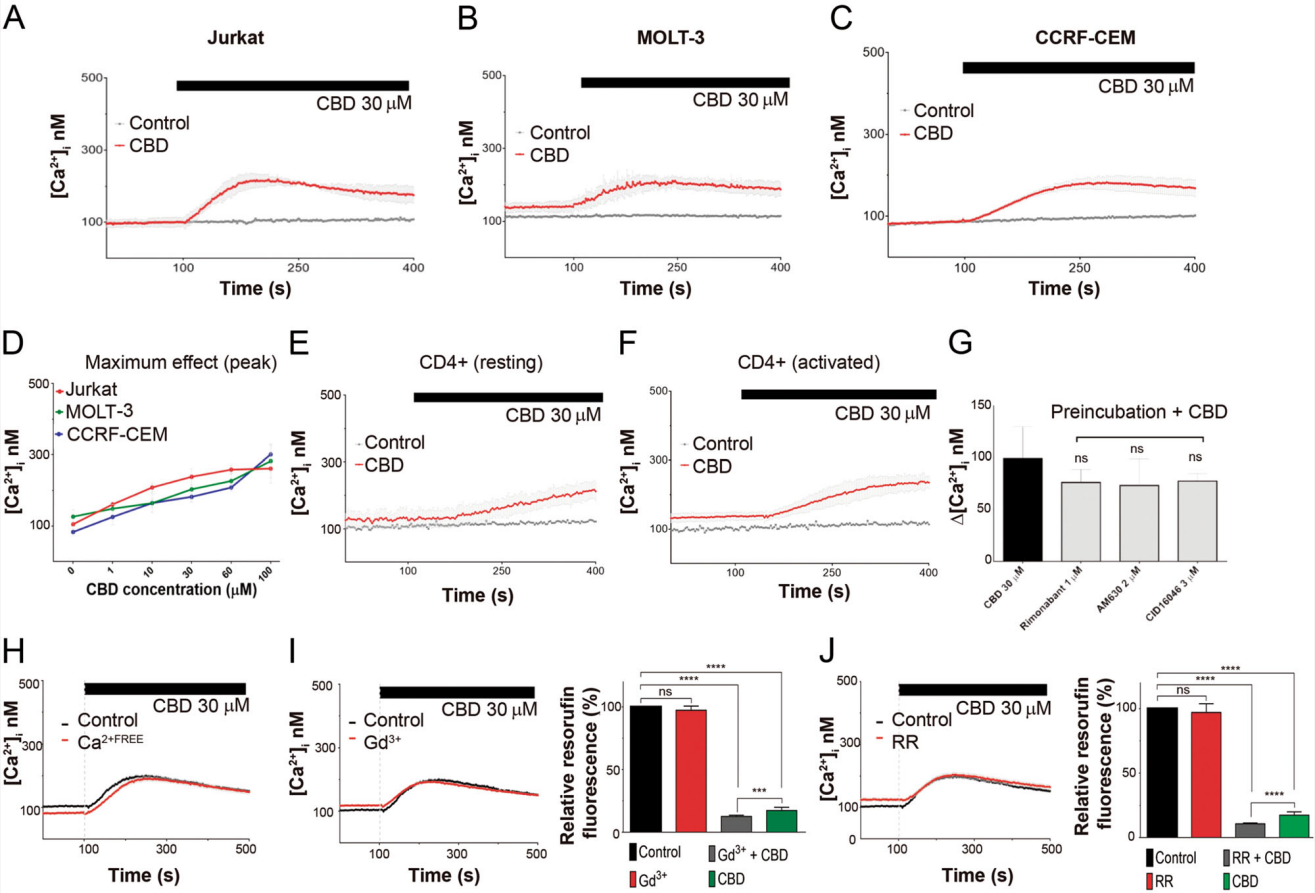
CBD induces cytosolic Ca^2+^ rise in human T-ALL cell lines and non-cancerous T cells. **a**–**c** [Ca^2+^]_i_ monitoring in leukemic cells, treated with CBD. Traces represent the mean ± SD of at least six samples from independent experiments. **d** Dose dependence of peak [Ca^2+^]_i_ values in T-ALL cell lines, exposed to CBD. Data are mean ± SD for at least six samples from independent experiments. **e**, **f** [Ca^2+^]_i_ monitoring in non-cancerous T cells in resting (**e**) and activated (**f**) states. Traces represent the mean ± SD of at least six samples from independent experiments. **g** Pharmacological analysis of [Ca^2+^]_i_ rise in response to CBD. Before CBD treatment, cells were preincubated over 20 min with either CB1 antagonist, rimonabant (1 μM), CB2 inverse agonist, AM630 (2 μM), or GPR55 antagonist, CID16020046 (3 μM); values Δ [Ca^2+^]_i_ were obtained by subtracting the [Ca^2+^]_i_ baseline level from [Ca^2+^]_i_ maximum increase. Data are mean ± SD of at least 6 samples from independent experiments. **h** [Ca^2+^]_i_ monitoring in Jurkat cells, suspended in Ca^2+^-free HBSS. Traces represent the mean ± SD of at least six samples from independent experiments. **i**, **j** Effect of Gd^3+^ and RR, non-selective blockers of plasma membrane Ca^2+^- permeable channels, on [Ca^2+^]_i_ (**i** and **j**, left) and cell viability evaluated by resazurin-based metabolic assay (**i** and **j**, right). Gd^3+^ (1 μM) or RR (1 μM) were added to Jurkat cells 20 min before the [Ca^2+^]_i_ measurement. **i**, **j** left: traces represent the mean ± SD of at least six samples from independent experiments. **i**, **j** right: data (resorufin fluorescence intensity) were normalized to vehicle-treated control and reported as mean ± SD (*n* = 8 of four independent experiments (****p < 0.0001, Student’s *t*-test)

CBD promoted a dose-dependent elevation of [Ca^2+^]_i_ from the resting level of 100 nM up to 300 nM, both in T-ALL cells (Fig. 5a–d) and in healthy lymphocytes (Fig. 5e, f). Pharmacological analysis revealed that neither CB1/2 nor GPR55 receptors were involved in the CBD-induced [Ca^2+^]_i_ rise. Importantly, CBD-induced [Ca^2+^]_i_ rise was neither dependent on Ca^2+^ permeable channels in plasma membrane nor on extracellular Ca^2+^ (Fig. 5g–j). Thus, the source of CBD-induced [Ca^2+^]_i_ rise were intracellular Ca^2+^ stores. Blockage of plasma membrane Ca^2+^ permeable channels by Gd^3+^ and ruthenium red (RR) did not protect leukemic cells against CBD (Fig. 5i, j).

**Fig. 6:**
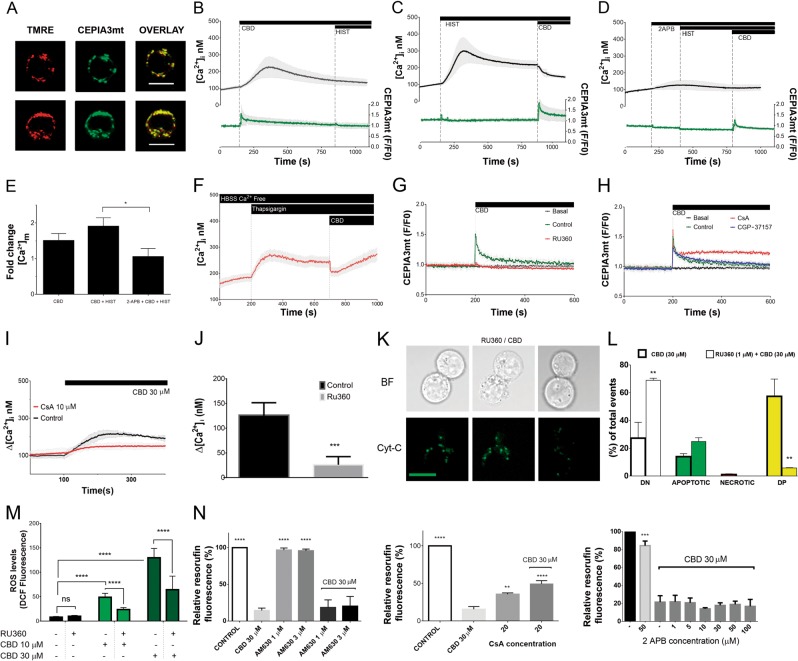
CBD directly induces mitochondrial Ca^2+^ overload and mPTP opening. **a** CEPIA3mt fluorescence is colocalized with mitochondrial marker TMRE in Jurkat cells. Scale bar: 10 μm. **b**–**d** Concurrent monitoring of [Ca^2+^]_i_ and [Ca^2+^]_m_ in Jurkat cells. [Ca^2+^]_i_ and [Ca^2+^]_m_ changes were evaluated with Fura-2 (2 μM) and CEPIA3mt, respectively. Note that cells were loaded either with Fura-2 or with CEPIA3mt; individual time courses for Fura-2 and CEPIA3mt, were synchronized with respect to the timepoint of CBD addition and averaged [Ca^2+^]_i_ and [Ca^2+^]_m_ responses were plotted at the upper and lower panels, respectively. CBD (30 μM), histamine (10 μM) and membrane-permeable IP_3_R blocker 2 APB (50 μM μM) were added as indicated. Traces are mean ± SD of at least six samples from independent experiments. **e** Peak values for [Ca^2+^]_m_ changes, induced by CBD, from the experiments shown in **b**–**d**, with a variable level of [Ca^2+^]_i_ due to manipulations with Ca^2+^ release from the ER. Bars represent mean ± SD of at least six samples from independent experiments. One-way ANOVA test (*p < 0.05). **f** [Ca^2+^]_i_ monitoring in Jurkat cells, loaded with Fura-2 (2 μM). ER Ca^2+^ was depleted by thapsigargin (1 μM). Experiments were performed in Ca^2+^-free medium (HBSS). Addition of CBD causes an abrupt decrease of [Ca^2+^]_i_ (*cf* with **c**). Traces are mean ± SD of at least three samples from independent experiments. **g**, **h** [Ca^2+^]_m_ monitoring in Jurkat cells, transfected with CEPIA3mt. CBD (30 μM) was added as indicated. When indicated, cells were preincubated over 20 min with either MCU blocker RU360 (1 μM), mPTP inhibitor CsA (10 μM), or inhibitor of mitochondrial Na^+^/Ca^2+^ exchanger NCLX CGP37157 (1 μM). Traces are mean ± SD of at least six samples from independent experiments. **i** [Ca^2+^]_i_ monitoring in Jurkat cells, loaded with Fura-2 (2 μM). CBD (30 μM) was added as indicated. Cells were preincubated during 20 min with vehicle or CsA (10 μM), specific inhibitor of the mPTP. Values Δ [Ca^2+^]_i_ were obtained by subtracting the [Ca^2+^]_i_ baseline level from the peak [Ca^2+^]_i_. Traces are ±SD of at least six samples from independent experiments. **j** Cytosolic Ca^2+^ response to CBD (30 μM) in Jurkat cells was abolished by a preincubation with the MCU blocker Ru360 (1 μM) over 20 min. Values Δ [Ca^2+^]_i_ were obtained by subtracting the [Ca^2+^]_i_ baseline level from peak [Ca^2+^]_i_. Data are mean ± SD of a minimum of six independent experiments (**p < 0.01; ***p < 0.001; Student’s *t*-test). **k** Representative images of Jurkat cells, transfected with EYFP-Cyt-C, pretreated with RU360 (1 μM, 20 min), and subsequently treated with CBD (30 μM, 1 h). Discrete green fluorescent puncta (pseudocolor) represent Cyt-C localization in intact mitochondria whereas Cyt-C release from mitochondria is evidenced by a more diffuse EYFP-Cyt-C distribution. Compare these images with Fig. 4d, **e** (vehicle- and CBD-treated cells) and note the protective effect of RU360. Scale bar: 10 μm. **l** MCU blocker RU360 effectively prevents CBD-induced cell death in Jurkat cells. Cell death was evaluated by flow cytometry, using Annexin V-AF488/PI double staining. Cells were preincubated with vehicle or RU360 (1 μM, 2 min), and then treated with CBD (30 μM, 6 h). Data of three independent experiments are present (**p < 0.01, one-way ANOVA test). **m** ROS levels were evaluated by DCF fluorescence intensity. Cells were either only treated with CBD (10 or 30 μM, 1 h, light and dark green bars, respectively) or additionally pretreated with RU360 (1 μM, 20 min). In all, 50 cells from at least three independent experiments were analyzed for each condition. Data are mean of ±SD. Statistic comparisons between control and CBD-treated samples, or between RU360-pretreated and non-pretreated samples were performed. ****p < 0.0001, one-way ANOVA. **n** Effects of the CB2 inverse agonist, AM630 (*n* = 8), mPTP inhibitor CsA (*n* = 6) and membrane-permeable IP_3_R blocker 2APB (*n* = 6) on the viability of Jurkat cells, treated with CBD. Cell viability was evaluated by resazurin-based metabolic assay (24 h). Data are mean ± SD. Statistical comparison was made in relation to CBD-treated samples; *p < 0.05; **p < 0.01; ***p < 0.001; ****p < 0.0001, one-way ANOVA

To monitor [Ca^2+^]_m_, Jurkat cells were transfected with a Ca^2+^-sensitive, mitochondrial targeted indicator CEPIA3mt (Kd = 11 μM)^28^. Specific mitochondrial targeting of CEPIA3mt was confirmed by its colocalization with TMRE (Fig. 6a). Concurrent measurement of [Ca^2+^]_i_ were conducted with cells loaded with a conventional cytosolic ratiometric dye Fura-2. CBD-induced [Ca^2+^]_i_ increase was preceded by [Ca^2+^]_m_ transient (Fig. 6b). Peak value for [Ca^2+^]_m_ signal was ~5 μM, basing on the titration curve for CEPIA3mt^28^_._ Notably, CBD-induced [Ca^2+^]_m_ transient occurred at time when [Ca^2+^]_i_ remained at the resting level. Thus, causal relation between CBD-induced [Ca^2+^]_m_ and [Ca^2+^]_i_ increases was further addressed. Jurkat cells highly express functional H1 histamine receptors and histamine treatment was proved to induce the inositol 1,4,5-trisphosphate receptor (IP_3_R)-dependent Ca^2+^ release from the ER for this cell model^29^. Artificial depletion of the ER Ca^2+^ by histamine or thapsigargin and block of the ER Ca^2+^ release via IP_3_R channels by 2-aminoethoxydiphenyl borate (2-APB) abolished the CBD-induced [Ca^2+^]_i_ increase. Thus, ER was the source of the CBD-induced [Ca^2+^]_i_ rise (Fig. 6c, d, f). CBD-induced [Ca^2+^]_m_ rise was potentiated by ER Ca^2+^ release (and concomitant cytosolic Ca^2+^ rise) and reduced upon the conditions, when ER Ca^2+^ release was blocked (Fig. 6c–e). Thus, ER Ca^2+^ release partly fuels the CBD-induced [Ca^2+^]_m_ rise. However, note that [Ca^2+^]_m_ rise was switched first upon CBD administration, not by Ca^2+^ release from the ER per se. Mitochondria act as a sink for Ca^2+^, artificially released from the ER prior to CBD application as evidenced by an abrupt decrease of [Ca^2+^]_i_ in a response to CBD (Fig. 6c, f).

Ca^2+^ uptake from the cytosol to mitochondria is mediated by voltage-dependent anion channel (VDAC) in the OMM and the mitochondrial Ca^2+^ uniporter (MCU) in the inner mitochondrial membrane (IMM). In our experiments, CBD-induced [Ca^2+^]_m_ rise was completely suppressed by a permeable MCU blocker Ru360 (Fig. 6g), but not significantly affected by CGP-37157, the inhibitor of the inner membrane Na^+^/Ca^2+^ exchanger, NCX (Fig. 6h). Specific inhibitor of mPTP, CsA, stabilized [Ca^2+^]_m_ at a high level (Fig. 6h). Of special note, the inhibition of mitochondrial Ca^2+^ overload and of mPTP by Ru360 and CsA, respectively, precluded the cytosolic Ca^2+^ rise (Fig. 6i–j). Thus, the ER Ca^2+^ release is causally dependent on the mitochondrial response. Collectively, our [Ca^2+^]_m_ data imply that CBD-induced Ca^2+^ uptake into the mitochondrial matrix via MCU caused a rapid [Ca^2+^]_m_ overload, which induced the mPTP opening and a subsequent [Ca^2+^]_m_ release. Thus, mitochondria appear to be a primary target for CBD and mPTP opening is required for the induction of Ca^2+^ release from the ER. MCU inhibitor Ru360 prevented CBD-induced ROS formation, Cyt-C release and cell death as revealed by Annexin V/PI assay (Fig. 6k–m). The last result is very essential, because it links mitochondrial Ca^2+^ overload to cell death. Remarkably, CsA, albeit per se possessing immunosuppressing activity, partially improved cell viability of CBD-treated cells, while CB2 inverse agonist AM630 and 2-APB were inefficient (Fig. 6n).

### CBD interacts directly with mitochondria to promote the organelle dysfunction

**Fig. 7:**
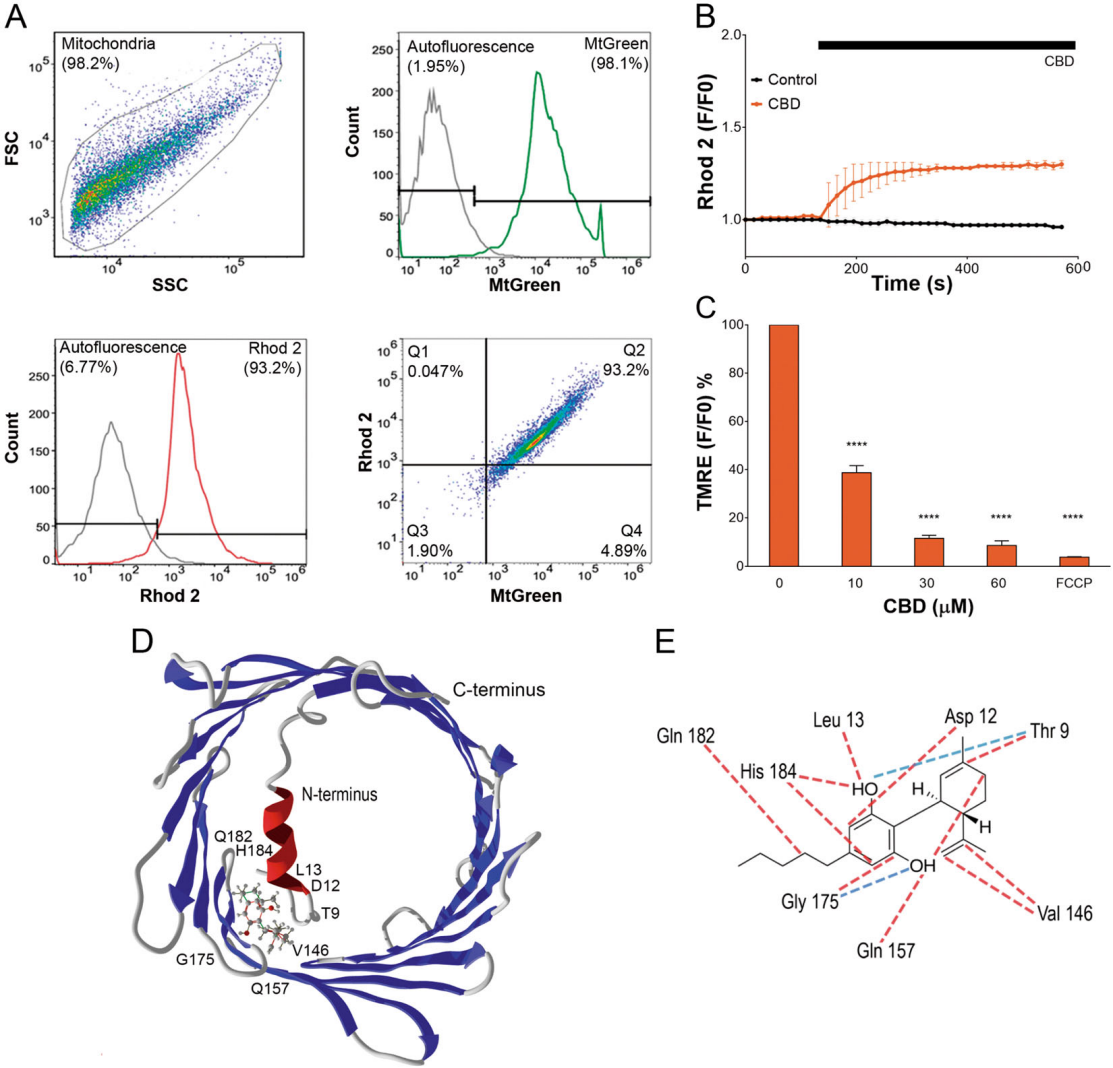
CBD directly interacts with mitochondria. **a** Isolation of mitochondria from Jurkat cells was corroborated by flow cytometry analysis. Representative experiment is shown. The purity of mitochondria population was determined as a percentage of double positive particles after staining (20 min) with MtGreen (200 nM) and Rhod-2 (2 μM). First, the mitochondria population was visualized in FSC/SSC dot blot (upper left panel). Non-stained mitochondria were used to determine the autofluorescence interval (gray histograms and corresponding intervals in upper right and lower left panels). Accordingly, positive intervals for MtGreen (green histogram and corresponding interval in upper right panel) and Rhod-2 (red histogram and corresponding interval in lower left panel) were determined. More than 90% of freshly isolated mitochondria were double-positive (lower right panel). Freshly isolated mitochondria were assayed immediately. **b** [Ca^2+^]_m_ uptake by freshly isolated mitochondria in a response to vehicle or CBD (30 μM) treatments. Traces are mean ± SD for three independent experiments. **c** CBD effect on membrane potential (Δψm) in isolated mitochondria. Freshly isolated mitochondria were stained with TMRE (400 nM) and exposed to different CBD concentrations for 10 min. FCCP was used as a positive control. Data are mean ± SD of at least three independent experiments (****p < 0.0001, one-way ANOVA test). **d** In silico analysis predicts that CBD interacts with VDAC1 at N-terminus and β9–11 residues. **e** Residues, interacting with CBD are depicted; hydrogen bonding interactions are colored in red, whereas steric interactions are depicted in blue

To verify the conclusion that mitochondria are direct targets for CBD action, mitochondria were freshly isolated from Jurkat cells. Mitochondria were stained with MtGreen and fluorescent Ca^2+^ indicator Rhod-2, and evaluated by flow cytometry. High percentage of double-stained particles evidenced high purity of mitochondria population (Fig. 7a). Upon exposure to 30 μM CBD isolated mitochondria exhibited [Ca^2+^]_m_ increase at external free Ca^2+^ of 100 nM, equal to the resting cytosolic Ca^2+^ level (Fig. 7b). The response of high affinity Ca^2+^ indicator Rhod-2 (Kd ~ 0.6 μM) was saturated, implying that free [Ca^2+^]_m_ level was above 1 μM for a long time. Incubation of isolated mitochondria with CBD over 10 min produced a strong dose-dependent decrease of ΔΨm (Fig. 7c), similar to that in whole cells (Fig. 4b).

### CBD binding to VDAC: in silico evidences

In some cell models CBD can induce cell death via direct interaction with VDAC, promoting its closure to a conformational substate^30^. This substate possesses a reduced capacity to transport metabolites and increased Ca^2+^ permeability^31^. We performed in silico analysis of VDAC- CBD interactions. VDAC surface and N-terminus were tested for CBD binding. Among the main VDAC cavities, β9–12 and N-terminus regions exhibited the most suitable sites for the CBD binding, basing on respective free energy changes (docking score, see Supplementary Table 1). 3D analysis of the CBD-VDAC interaction revealed that more likely CBD interacts with 3 residues, Thr9, Asp12, and Leu13 at the N-terminus and neighboring pore residues Val146, Gln157, Gly175, Gln182, and His184 (Fig. 7d**)**. Further analysis revealed that CBD binding is stabilized mainly by steric interactions and hydrogen bonds (Fig. 7e). Of putative CBD-interacting residues, Thr9, Asp 12, and Hist184 have a highest contribution to the overall binding energy (Supplementary Fig. 1, Supplementary Table 1).

## Discussion

A crucial role of mitochondria in cell metabolism and bioenergetics, as well as in signaling pathways, regulation of transcriptional activity, proliferation, migration, and cell death is tightly related to their involvement into the intracellular Ca^2+^ dynamics^32^. In the present study, by means of concurrent monitoring of [Ca^2+^]_m_ and [Ca^2+^]_i_ in a response to the CBD treatment, we have provided the experimental evidences that mitochondria are primary CBD target in T-ALL. In the first place, acute transient [Ca^2+^]_m_ rise, preceding the [Ca^2+^]_i_ increase, was observed in a response to CBD (Fig. 6b). Furthermore, [Ca^2+^]_m_ increase together with a dissipation of ΔΨm was observed also in isolated mitochondria, treated with CBD (Fig. 7b, c). Ca^2+^ accumulation in mitochondrial matrix requires the crossing of both OMM and IMM. VDAC channel, ubiquitously expressed in OMM, is normally responsible for the OMM permeability to Ca^2+^^33^. Under physiological conditions, VDAC permeability to Ca^2+^ at levels, required for an optimal function of TCA enzymes, is regulated by VDAC interaction with IP_3_R in ER and chaperone 75 kDa glucose-regulated protein GRP75^34^. Our in silico analysis which demonstrated that CBD may interact directly with VDAC (Fig. 7) is in agreement with findings by others that CBD colocalizes with VDAC-rich mitochondrial membranes fraction from BV-2 microglia cell line and that purified VDAC, incorporated into lipid bilayers, is switched from fully open to major subconductance state by CBD^30^. Such “closed” channel state is known to be highly permeable for Ca^2+^, due to the exposure of anionic groups within the channel pore^31^.

**Fig. 8:**
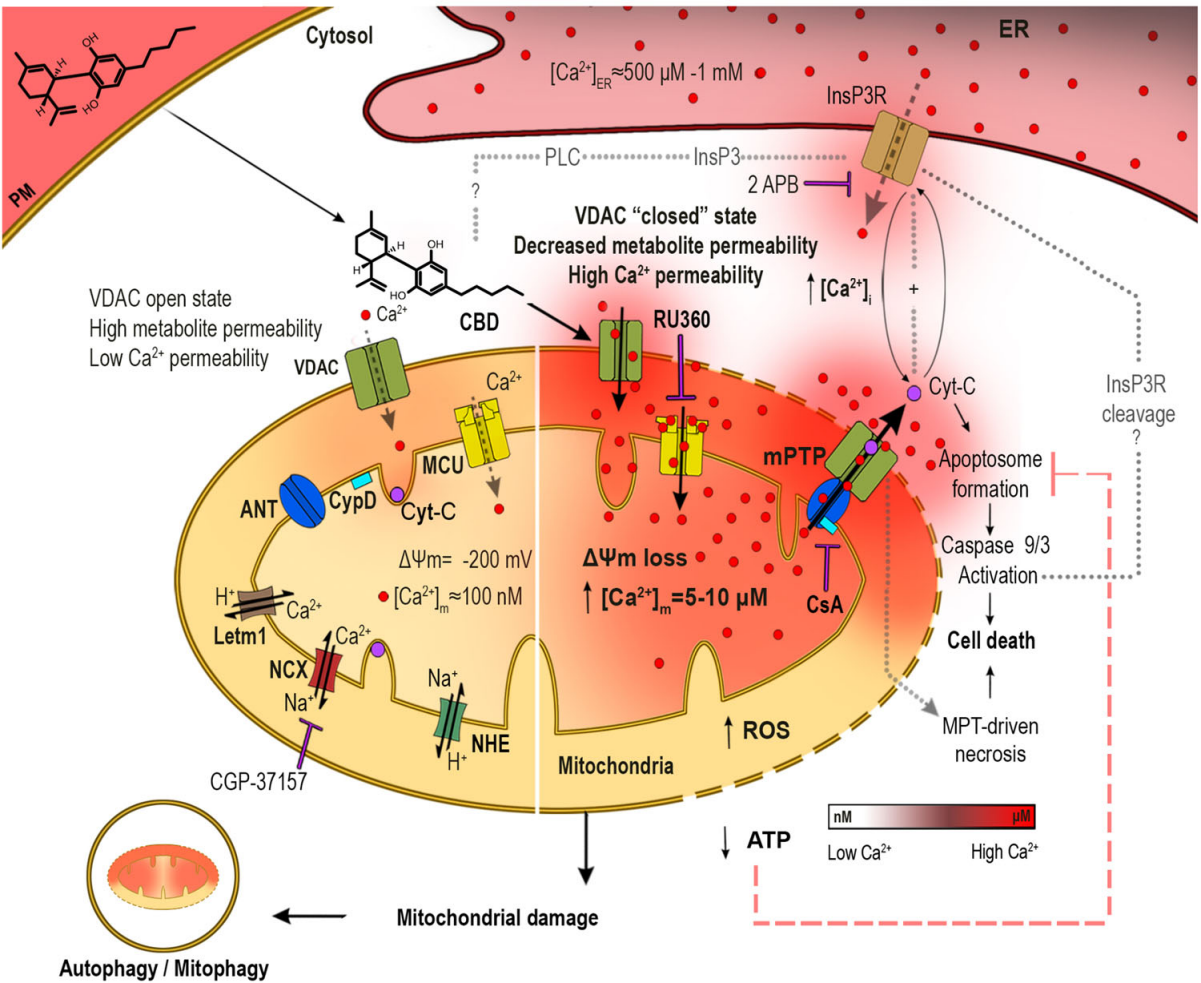
Proposed mechanism for the CBD effect on T-ALL cells. Highly lipophilic CBD readily permeates plasma membrane and enters the cytosol, approaching mitochondria. Direct CBD interaction with VDAC favors the channel closed substate with increased Ca^2+^ permeability. It favors mitochondrial Ca^2+^ uptake through VDAC and MCU, leading to the mitochondrial Ca^2+^ overload that promotes the mPTP formation, Δψm loss, mitochondrial swelling, and cristae disruption. mPTP opening promotes the Cyt-C release from mitochondria. In cytoplasm, Cyt-C may orchestrate the apoptosome formation, caspases´ activation, and triggers the intrinsic apoptosis. mPTP rapidly triggers Ca^2+^ release from the ER, which tends to promote the mitochondrial Ca^2+^ overload in a feedforward manner. The CBD-induced dysfunction of mitochondria is accompanied by severe oxidative stress and rapid loss of ATP production, resulting in the MPT-driven necrosis. Autophagy occurs in T-ALL cells treated with sublethal CBD concentrations

Based on the available data, the following working model can be proposed (Fig. 8). Due to its direct interaction with CBD, VDAC1 is “fixed” in the Ca^2+^-permeable state. This causes Ca^2+^ entry into the intermembrane space. Ca^2+^ needs to concentrate to micromolar level there, to unlock and activate the MCU^35^. This is commonly believed to be induced by a substantial increase of extramitochondrial Ca^2+^. Our data suggest that mitochondrial Ca^2+^ overload occurred already at resting 0.1 μM cytosolic Ca^2+^ (Figs. 6b, d and 7b). The driving force for Ca^2+^ accumulation within the intermembrane space could be a negative Donnan potential of −20 to −40 mV across the OMM^36^. This allows the concentration of Ca^2+^ in the intermembrane space up to 20-fold as compared to the cytosolic Ca^2+^ concentration, providing that the outer membrane is permeable for Ca^2+^. The magnitude of the Donnan potential across the OMM depends on the accumulation of impermeable large anions within the intermembrane space. Such accumulation, e.g. of ATP, may be provoked by the same conformational shift within VDAC1, which not only increases its Ca^2+^ permeability, but also makes it adenine nucleotide-impermeable^36^. Activation of MCU along with a large negative potential across the IMM drives Ca^2+^ entry into the mitochondrial matrix, leading to a rapid [Ca^2+^]_m_ overload (Fig. 6). The latter triggers a stable mPTP formation, which causes depolarization of the IMM, mitochondrial dysfunction, severe oxidative stress, and Ca^2+^ and Cyt-C release into the cytosol.

Noteworthy, multiple leukemic phenotypes express higher levels of VDAC in a comparison to healthy cells and VDAC expression is increased upon chemotherapy. Moreover, the degree of overexpression is positively correlated with the cell death induction by anticancer agents^37–39^.

We have observed different outcome of the CBD treatment depending on its concentration. At high CBD concentration, various scenarios of regulated cell death may be triggered in LLA-T (Fig. 8). In the present study, we reported apoptotic features such as Cyt-C release from mitochondria to cytosol, activation of caspases 9/3 and externalization of phosphatidylserine. On the other hand, appearance of the huge Annexin V^+^PI^+^ population in the early phases of CBD treatment evidenced MPT-driven necrosis. One can hypothesize that development of MPT-driven apoptosis may be blocked due to energy penalty, a decreased ATP level caused by a cessation of oxidative phosphorylation by defunct mitochondria^40^. At sublethal (10 μM CBD) concentration, autophagy was induced and apparently efficiently prevented cell death (Fig. 1b, c, g, h; Fig. 3a–d). Low CBD concentrations even stimulated cell proliferation (Fig. 1b). This phenomenon may be explained in the frame of the same basic model, providing they promoted only limited [Ca^2+^]_m_ increase. Such moderate [Ca^2+^]_m_ accelerates metabolism due to Ca^2+^-dependence of TCA cycle enzymes^41^.

In conclusion, CBD directly targets mitochondria in T-ALL and changes their capacity to handle Ca^2+^, which in turn affects multiple cellular functions, including ROS production and Ca^2+^ signaling, metabolic switch and the induction of autophagy and cell death. The latter is directly proved for our experimental model as the inhibitor of mitochondrial Ca^2+^ uptake Ru360 protected T-ALL cells from the CBD-induced cell death. Considering the pivotal role of mitochondria in oncogenic re-programming, CBD may be plausible candidate to be included into chemotherapeutic protocols. Importantly, resting T cells, representing major T lymphocyte population, were resistant to CBD and retained their ability to antigen activation. Healthy activated T cells were CBD-sensitive, but this population is small in T-ALL patients with a weakened immunologic system. However, contrasting effects of low and high CBD concentrations and possible differences in its tissue distribution and bioavailability requires further studies on animal models, with a focus on safety issues.

## Materials/subjects and methods

### Cell lines and culture conditions

Leukemic cell lines Jurkat (ATCC®TIB™, Clone E6-1, male, 14 years), MOLT-3 (ATCC®CRL-1552™, male, 19 years), CCFR-CEM (ATCC®CCL-119™, T-ALL, female, 4 years), K562 (ATCC®CCL-243™, female, 53 years), Reh (ATCC®CRL-8286™, female) and RS4;11 (ATCC®CRL-1873™, female, 32 years) purchased from ATCC® (Manassas, VA, USA) were grown in suspension in Advanced RPMI 1640 medium, supplemented with 5% (v/v) of heat-inactivated fetal bovine serum (FBS), 100 U/mL of penicillin, 100 µg/mL streptomycin and 1% of GlutaMAX™ (all from Invitrogene, Carlsbad, CA, USA). Tandem-labeled mCherry-GFP-LC3 Jurkat cells (gift from Dr. A. Thorburn, Colorado, USA) were maintained in complete growth medium (as for wild type Jurkat cells), additionally supplemented with 400 µg/mL of hygromycin B (Sigma, H3274) for selection. Suspension cells were maintained in the logarithmic growth phase by daily medium refreshment. Adherent cell lines MDA-MB-231 (ATCC®HTB-26™, female, 51 years), MCF7 (ATCC®HTB-22™, female, 69 years), SiHa (ATCC®HTB-35™, female, 55 years) and HeLa (ATCC®CCL-2™, female, 31 years) were maintained in DMEM medium supplemented with 10% (v/v) of heat-inactivated FBS, 100 U/mL of penicillin, 100 µg/mL streptomycin and 1% of GlutaMAX™. Adherent cells were passaged when they were in the logarithmic growth phase. All cells were cultured in a humidified incubator in 5% CO_2_/95% air atmosphere at 37 °C.

### Human samples

Blood samples (10 mL) from non-cancerous, apparently healthy volunteers (6 males and 6 females, younger than 35 years) were collected by capacitated personal under aseptic conditions. A written informed consent was obtained from all persons, prior to sample collection, according to the Declaration of Helsinki. Sample acquisition protocol was evaluated and approved by the Bioethics and Biosecurity Committee of the Biomedical Research Centre and the Faculty of Medicine of the University of Colima, in agreement with the federal laws (Artículo 100, Ley General de Salud).

### Cannabidiol

CBD solution in methanol (10 mg/mL, equivalent to 32 mM) was purchased from Cayman Chemical (90081) and stored at −20 °C. Working solutions in complete growth medium were prepared daily. Methanol (154903, Merck) was used in vehicle-treated controls. Reference methanol concentration for a vehicle control was 0.3% v/v, which corresponds to that applied with the highest tested CBD concentration (100 μM).

### Purification and activation of CD4^+^ lymphocytes

Heparinized freshly isolated blood samples were diluted 1:1 with cold PBS. Peripheral blood mononuclear cells (PBMC) were separated by centrifugation in Ficoll (17-144002, Ficoll-Paque 1.073, GE Healthcare) gradient (1:1.5 blood/Ficoll ratio, 1000 × *g*, 30 min, RT). PBMC were collected from the interphase and carefully washed in PBS. Finally, cells were resuspended in a fresh RPMI 1640 medium, supplemented with 10% of FBS and incubated overnight for cell recovery. Next day, PBMC were subjected to negative selection (to avoid activation) with human CD4^+^ T cell isolation kit (130-096-533, Miltenyi Biotec Miltenyi Biotec), following manufacturers’ specifications. Briefly, PBMC were collected, washed (400 × *g*, 5 min) and the pellet was resuspended in cold MACS buffer. Live cells were counted (trypan blue exclusion test) and incubated with CD4^+^ T cell biotin-antibody cocktail (1 μL/1 × 10^6^ cells) against CD8a, CD11b, CD11c, CD19, CD45R (B220), CD49b (DX5), CD105, Anti-MHC Class II, Ter-119, and TCRγ/δ, for 20 min at 4 °C with agitation, followed by incubation with microbeads, conjugated to monoclonal anti- biotin antibodies (2 μL/1 × 10^6^ cells) over 20 min at 4 °C. Next, 1 mL of MACS buffer was added, cells were centrifuged (400 × *g*, 5 min), supernatant was discarded to remove the excess of antibodies and cells were resuspended in 1 mL of cold MACS buffer. CD4^+^ T cell population was separated using a MACS separator. LS column was placed in the MAC separator, rinsed with 3 mL of MACS buffer and cell suspension was added. Enriched CD4^+^ T (negative) cells were collected and column was washed three times with MACS buffer for a complete CD4^+^ harvesting. Enriched CD4^+^ T cell population was centrifuged (400 × *g*, 5 min), resuspended in complete Advanced RPMI 1640 medium and incubated at 37 °C in a humidified atmosphere (5% CO_2_, 95% air) prior to the experiments. The population purity was more than 95% as verified by flow cytometry (FACSCanto II, BD Biosciences), using antiCD4 antibodies (BioLegend 357404).

For polyclonal activation, resting CD4^+^ lymphocytes were pretreated in 96-well plates with antiCD3 monoclonal antibodies (5 µg/mL) (BD, 555336) for 2 h at 37 °C. Medium excess was removed and cells were further incubated with antiCD28 monoclonal antibodies (2 µg/mL) (BD, 555725) for 4 days. Ligation of CD3/CD28 provides an antigen-independent activation stimulus by cross-linking T cell receptor (TCR), resulting in transit from quiescent to proliferation state.

### Resazurin-based metabolic assay

To estimate cell drug toxicity, resazurin-based metabolic assay was used. Bioreduction of resazurin reagent (Tox 8, Sigma-Aldrich) by viable cells reduces the amount of its oxidized form and concomitantly increases the amount of its fluorescent intermediate resorufin. The amount of dye conversion in solution was measured fluorometrically, using a fluorescence plate reader GloMax Discover (PROMEGA). Cells (10^6^/mL) were seeded into 96-well plates in 180 µL of complete RPMI medium per well. Cells were incubated 24 h without or with CBD (1–100 μM). For assay, 20 μL aliquots of resazurin reagent were added to each well to a final volume of 200 μL and cells were further incubated for 4 h (37 °C). Samples’ fluorescence was measured by excitation at 520 nm and emission was collected at 580–640 nm. RPMI fluorescence was subtracted for each condition. Samples were run in triplicate, in at least three independent experiments. Data obtained from resorufin fluorescence were averaged, normalized to their controls and expressed as cell viability.

### Sequential CBD administration

Jurkat cells (2.5 × 10^5^/mL per well) were seeded into a 48-well plate in complete Advanced RPMI medium. Cells were exposed to CBD (1–100 µM, dissolved in 500 μL of RPMI) and incubated for 24 h. After incubation, cell culture was gently resuspended, and 10 μL of suspension was taken for cell counting, using a hemocytometer and trypan blue exclusion test, to determine the number of viable cells. A second dose of CBD was administered, and cells were incubated for the next 24 h. The procedure was repeated one more time, so that total assay was completed in 72 h. Initial cell number was taken as 100%, and daily cell count was normalized to this point. Three cell counts (*n* = 3) from three independent experiments were averaged and expressed as percentage of viability.

### CD4^+^ T cell recovery test

Non-cancerous CD4^+^ T-cells from healthy donors were cultured (1 × 10^6^ cells/mL) in complete Advanced RPMI 1640 medium. Cells were preincubated with CBD (30 μM) or vehicle over 24 h. After CBD treatment, cells were washed and resuspended in a CBD-free medium and used for activation with antiCD3 and antiCD28 antibodies, as described earlier. Cell viability was determined by resazurin-based metabolic assay (Tox8, Sigma-Aldrich) as previously described, using a fluorescence plate reader (GloMax Discover, PROMEGA). Samples were excited at 520 nm and emission was collected at 580–640 nm. RPMI fluorescence was subtracted for each condition and data was generated in triplicate, in at least three independent experiments. Data obtained from resorufin fluorescence were averaged, normalized to their control values and expressed as cell viability.

### Migration assay

Leukemic cells were seeded in 12-well plates (3422, Transwell system, Corning Inc.) and preincubated for 1 h with CBD. Pretreated cells (2 × 10^5^) were placed in 400 µL of serum-free medium in the upper chamber of Transwell inserts (8 μM pore size). Recombinant human CXCL12 (Sigma-Aldrich) was used as a chemoattractant (100 ng/mL) in the lower chamber, filled with complete RPMI (10% FBS) medium. Cells were allowed to migrate over 4 hours. After incubation, insert was removed and cells from the lower chamber were counted, using hemocytometer. Migration was expressed as a percentage of migrated cells in relation to the total cell number.

### Cell death analysis

For this assay, Alexa Fluor® 488 Annexin V/Dead Cell Apoptosis Kit (V13241, Thermo Fisher Scientific) was used, following manufacturer specifications. Kit provides a nucleophilic marker (propidium iodide, PI, Ex/Em max = 535/617 nm) as an indicator of plasma membrane damage and Annexin V-Alexa Fluor 488 (Ex/Em max = 488/510 nm), binding to externalized phosphatidylserine (a hallmark of apoptosis). Jurkat cells (10^6^/mL) were seeded in a 24 well plate and incubated with or without CBD (0–100 μM) for determined period (2, 4, 6, or 12 h). After the incubation period, cells were centrifuged (400 × *g*) and washed with cold PBS. Then cells (1 × 10^6^) were resuspended in 100 μL of 1X Annexin V-binding buffer and 5 μL of Annexin V conjugate with 1 μL of PI working solution (200 μg/mL) were added. The mixture was incubated for 20 min at room temperature (protected from light), 200 μL of Annexin V - binding buffer was added and cells were analyzed either by confocal microscopy (LSM 700, Carl Zeiss) or by flow cytometry (FACSCanto II, BD Biosciences).

For confocal microscopy, cells were placed into home-made coverslips-bottomed chambers (poly-l-lysine-coated coverslips were fixed at the bottom of chamber using Dow corning® high vacuum grease). 40×/63x oil-immersion objectives were used. For excitation, 488 nm laser was used for both Alexa Fluor 488 and PI. Raw data were further processed, and images were generated using Zen software (Zeiss).

For flow cytometry analysis, color compensation (Alexa Fluor 488 vs PI) was performed previously to data acquisition. 488 nm laser was used for excitation. PI fluorescence was measured using 556LP mirror and 585/42 filter, Alexa Fluor 488 fluorescence was measured using 502LP mirror and 530/30 filter. Debris and doublets were gated out, and 10,000 events of single cells per sample were collected. Autofluorescence control was used to determine the positive fluorescence threshold. Annexin V^−^PI^−^ populations were classified as healthy, Annexin V^+^PI^−^ as early apoptotic, Annexin V^-^PI^+^ as primary necrotic, and Annexin V^+^PI^+^ as necrotic/late apoptotic. Data analysis was performed with FlowJo 10.2 software.

### Determination of mitochondrial membrane potential

Jurkat cells (10^6^/mL) were double-labeled with MitoTracker^TM^ Green (100 nM, Ex/Em max = 490/518 nm; M7514, Thermo Fisher Scientific) and TMRE (200 nM, Ex/Em max = 555/582 nm; T669, Thermo Fisher Scientific), both from Thermo Fisher Scientific, by incubating over 30 min. After incubation period, cells were centrifuged (400 × *g*, 10 min) and washed with Hanks Balanced Salt Solution (HBSS; NaCl 143 mM, KCl 6 mM, MgSO_4_ 5 mM, CaCl_2_ 1 mM, HEPES 20 mM, BSA 0.1%, glucose 5 mM, pH 7.4, ≈300 mOsm) to remove excessive dye. For imaging, cells were placed in home-made coverslips-bottomed chambers and analyzed by confocal microscopy (LSM 700, Carl Zeiss) in a time series mode. Images were acquired every second and raw data were further processed by ImageJ program, where regions of interest (ROI) were defined, based on MitoTracker Green distribution. The fluorescence of each ROI for both dyes (TMRE/MtGr) was then averaged and expressed as a temporal ratio between the fluorescence of each frame and the initial fluorescence (F/F0). To evaluate drugs’ effect, CBD (0–100 μM) or FCCP (10 μM; C2920, Merck) were administered, and cells were incubated for 10 min, centrifuged (400 × *g*, 10 min), resuspended in HBSS, and transferred to a 96-well plate. TMRE retention was assessed by measurement of fluorescence intensity, using a GloMax Discover plate reader, by exciting the sample at 549 nm and collecting the emission signal at 575 nm. Data from independent experiments were averaged and the effect of CBD/FCCP was expressed as percentage of TMRE signal in comparison to control.

### EYFP-Cyt c transfection

DH5α competent bacteria (18258012, Thermo Fisher Scientific) were transformed by heat shock and EYFP-Cyt-C construct^42^ was added and incubated for 14 h at 37 °C in LB agar (22700025, Thermo Fisher Scientific), supplemented with 100 μg/μL of ampicillin (11593027, Thermo Fisher Scientific). Colonies were selected and transferred to supplemented media for further incubation during 14 h for bacterial growth. Plasmid DNA was purified by NucleoBond XtraMidi (740410.10, Macherey-Nagel) kit, and DNA purity and concentration were evaluated spectrophotometrically by absorption at 260/280 nm. For transfection, 10^5^ Jurkat cells were starved in Optimem reduced media for 12 h, then exposed to complexes composed by lipofectamine 3000 (L3000015, Thermo Fisher Scientific) and plasmidic DNA (500 ng) and centrifuged (400 × *g*) for 30 min to promote interaction. Transfected cells were incubated at 37 °C, with 5% CO_2_ overnight, whereas FBS (10%) was added at the next day. Protein expression was monitored, and experiments were performed 12 h after transfection. Images were acquired using a confocal microscope (LSM 700, Carl Zeiss) equipped with ×40/×63 oil-immersion objectives.

### Cyt-C release microscopic assay

Jurkat cells transfected with EYFP-Cyt-C (Ex/Em max = 514/526 nm) were labeled with TMRE (200 nM, 30 min) to confirm mitochondrial EYFP-Cyt-C localization. EYFP-Cyt-C expression and distribution were evaluated in Jurkat cells 12 h after transfection by confocal microscopy. The effect of CBD (30 μM) was monitored in transfected cells incubated for indicated period with the drug. To evaluate the effects of CsA and Ru360, cells were preincubated with one of these drugs for 10 min, followed by treatment with CBD. For data acquisition, cells were placed in home-made coverslips-bottomed chambers. The images were acquired using a confocal microscope (LSM 700, Carl Zeiss), equipped with ×40/×63 oil-immersion objectives.

### ROS production

To evaluate ROS production, 2′,7′-Dichlorofluorescin diacetate (DCFDA, D6883, Merck) was used following manufacturer’s recommendations. Jurkat cells (10^6^/mL) were loaded with permeable DCFDA (2 μM) for 30 min in HBSS. After this, cells were washed to remove extracellular dye. Cells were resuspended in RPMI1640 medium and allowed to recover and to hydrolyze the AM groups, producing the insoluble form. Culture media was removed, and cells were resuspended in HBSS and treated either with PMA (1 μM, P8139, Merck) as a positive control or with CBD (10 and 30 μM). For data acquisition, cells were placed in home-made coverslips-bottomed chambers. Images were acquired by confocal microscopy (LSM 700, Carl Zeiss), equipped with ×40/×63 oil-immersion objectives and analyzed by ZEN imaging software.

### Caspase 3/9 assay

Caspase 3/9 colorimetric assays (K106/119, BioVision) were used following manufacturer’s recommendations. Jurkat cells were grown in the presence of vehicle, staurosporine (positive control) or CBD during 12 h. After this period, 10^6^ cells from each sample were washed, resuspended in lysis buffer and incubated over 10 min on ice. Then samples were centrifuged (10,000 × *g*, 15 min) and supernatants (cytosolic fraction) were transferred into new tubes. For caspase activity assays, 100 μg of protein (as estimated by BCA protein quantification kit, Merck) from every sample were mixed with 50 μL of reaction buffer (containing 10 mM DTT) and 5 μL (4 mM) of the caspases´ substrates (DEVD-*p*NA for caspase 3, LEHD-*p*NA for caspase 9). Mixtures were incubated for 2 h at 37 °C and placed into a 96-well plate. Absorbance was measured at 405 nm, using a GloMax Discover plate reader. Data from three independent experiments were averaged and normalized in relation to non-treated cells, yielding fold increase in caspase activity.

### Transmission electron microscopy

Jurkat cells were treated with CBD (30 μM) for 2 h. Next, control or treated cells were centrifuged (400 × *g*), supernatants were discarded and the pellets were fixed with 2.5% glutaraldehyde and post-fixed in 1% OsO_4_ and 0.8% K_4_Fe(CN)_6_ • 3H_2_O, and 5 mM Ca^2+^. Post-fixed cells were dehydrated in acetone and embedded in Epon. Ultrathin sections were stained with uranyl acetate and lead citrate and examined under a JEOL JEM 12 000 EII transmission electron microscope at the Unidad de Imagenología, Instituto de Fisiología Celular (IFC), UNAM, Mexico City.

### CEPIA3mt transfection

DH5α competent bacteria (18258012, Thermo Fisher Scientific) were transformed by heat shock and CEPIA3mt/pCMV construct (58219, Addgene) was added^27^. Samples were incubated for 14 h at 37 °C in LB agar (22700025, Thermo Fisher Scientific), supplemented with 100 μg/μl of ampicillin (11593027, Gibco). Colonies were selected and transferred to supplemented media for further incubation during 14 h for bacterial growth. Plasmid DNA was purified by NucleoBond XtraMidi (740410.10, Macherey-Nagel) kit and purity and DNA concentration were determined by spectrophotometry (absorption at 260/280 nm). For the transfection, 10^5^ Jurkat cells were starved in Optimem reduced media over 12 h, collected, exposed to complexes of lipofectamine 3000 (L3000015, Thermo Fisher Scientific) and plasmidic DNA (1 µg), and centrifuged (400 × *g*) for 30 min to promote interaction. Cells were incubated at 37 °C with 5% CO_2_ overnight, whereas FBS (10%) was added at the next day. Protein expression was monitored, and experiments were performed 24 h after transfection. For confocal imaging, cells were placed in home-made coverslips-bottomed chambers. Images were acquired using a confocal microscope (LSM 700, Carl Zeiss) equipped with ×40/×63 oil-immersion objectives.

### Determination of the intracellular free Ca^2+^ concentration

For free [Ca^2+^]_i_ measurements, cells were loaded with ratiometric Ca^2+^ indicator Fura-2 (F1201, Thermo Fisher Scientific). Cells were twice washed with PBS and resuspended in a loading solution (HBSS, 0.01% pluronic acid, 2 µM Fura-2/AM), incubated for 30 min at room temperature protected from light, washed, and resuspended in HBSS. Changes in fluorescence were recorded with a F7000 spectrophotometer (Hitachi High-Technologies). Measurements were realized in quartz cuvettes, containing 1.5 × 10^6^ cells/mL. Loaded cells were excited alternately at 340/380 nm and the fluorescence emission was collected every 2 s at 510 nm. Fluorescence was recorded by means of the FL-solutions software. Maximum and minimum free [Ca^2+^] levels were determined at the end of each experiment by adding 0.3% Triton X-100 and consequent addition of EGTA to a final concentration of 35 mM, respectively. Free [Ca^2+^]_i_ was calculated by using the following equation:

$$\left[ {{\mathrm{Ca}}^{2 + }} \right]_{\mathrm{i}}\; = K_{\mathrm{d}}\left( {R - R_{{\mathrm{MIN}}}} \right)/\left( {R_{{\mathrm{MAX}}} - R} \right)$$

where *R* stays for the ratio of fluorescence intensity upon excitation at 340 to that at 380 nm and *R*_MIN_ and *R*_MAX_ correspond to maximal and minimal values of this ratio, determined as described above^43^.

In some experiments Ca^2+^-free HBSS was used (NaCl 143 mM, KCl 6 mM, MgSO_4_ 5 mM, HEPES 20 mM, BSA 0.1%, glucose 5 mM, EGTA 1 mM, pH 7.4, ≈300 mOsm).

### Mitochondrial Ca^2^^+^ measurements

CEPIA3mt (Ex/Em max = 488/510 nm) expression and its mitochondrial localization were confirmed in transfected Jurkat cells, loaded with TMRE, by confocal microscopy (LSM700, Carl Zeiss). For [Ca^2+^]_m_ measurements, 10^5^ cells (24 h after transfection) were placed in a quartz cuvette and fluorescence was evaluated by spectrofluorometry, using a F7000 HITACHI spectrofluorometer (FL solution software). Samples were excited at 488 nm and fluorescence was measured at 510 nm. [Ca^2+^]_m_ was evaluated as fluorescence intensity in relation to the initial fluorescence intensity (F/F0).

### Mitochondria isolation

Mitochondria were isolated as reported earlier^39^, with some modifications. Jurkat cells were harvested and centrifuged (400 × *g*) at 4 °C for 10 min, washed twice with PBS (pH 7.4) and resuspended in 10x of volume of isolation buffer (IB, HEPES-KOH 20 mM, PMSF 1 mM, sucrose 250 mM, mercaptoethanol 1 mM, EGTA 1 mM, EDTA 1 mM, MgCl_2_ 1.5 mM, KCl 10 mM). Cells were incubated on ice for 10 min and homogenized with a Dounce homogenizer. Homogenate was then centrifuged at 650 × *g* for 10 min at 4 °C, pellet, containing nuclei, was discarded and the supernatant was collected for a further 12500 × *g* centrifugation for 30 min at 4 °C. Pellet containing the heavy membrane fractions (HMF) was collected and the supernatant was discarded. HMF were washed with IB and resuspended in isotonic sucrose buffer (sucrose 250 mM, EDTA 1 mM, Tris-HCl pH 7.4 10 mM). Homogenate was placed in a discontinuous sucrose gradient (sucrose 1/1.5 M, EDTA 1 mM, Tris-HCl pH 7.4 10 mM) and centrifuged for 25 min at 60,000 × *g*, 4 °C. Mitochondria were collected from the interphase, washed and resuspended in the experimental buffer (KCl 125 mM, KH_2_PO_4_ 1 mM, Tris-HCl 10 mM, glutamate 5 mM, malate 2.5 mM, EGTA 1 mM, CaCl_2_ 0.7 mM). Isolated mitochondria were suspended in the lysis buffer (Nonidet P-40 0.5% w/v, Tris-HCL 50 mM, NaCl 150 mM, EDTA 1 mM, and PMSM 1 mM) for 1 h and centrifuged at 15000 × *g* for 5 min. The supernatant was collected, and isolation yield was estimated by the protein content (BCA quantification assay). Finally, mitochondrial samples, containing 50 µg of protein, were used in the experiments. To evaluate the purity and integrity of isolated mitochondria, a small fraction was stained with MtGreen (200 nM, Ex/Em max = 490/510 nm; M7514, Thermo Fisher Scientific) as mitochondrial marker, followed by staining with Rhod (2 μM, Ex/Em max = 552/581 nm; R1244, Thermo Fisher Scientific) or TMRE (200 nM, Ex/Em max = 549/575 nm; T669, Thermo Fisher Scientific). Samples were acquired by flow cytometry (FACSCantoII, BD Biosciences) and data were analyzed by FlowJo software.

### Ca^2+^ measurement in isolated mitochondria

Freshly isolated mitochondrial samples (50 μg of protein per sample) were incubated with Rhod2 (2 μM) over 30 min, washed by centrifugation (12500 × *g*, 5 min) and resuspended in experimental buffer (KCl 125 mM, KH_2_PO_4_ 1 mM, TrisHCl 10 mM, glutamate 5 mM, malate 2.5 mM, EGTA 1 mM, CaCl_2_ 0.7 mM). Samples were placed in a quartz cuvette, and fluorescence was evaluated by F7000 spectrophotometer (Hitachi High-Technologies), using excitation at 552 nm and collecting the fluorescence at 581 nm. Data was recorded using the FL-Solution software (Hitachi). [Ca^2+^]_m_ change was evaluated by taking fluorescence for each acquisition in relation to the initial fluorescence value, F/F0.

### Membrane potential measurements on isolated mitochondria

Isolated mitochondria (50 μg of protein/sample) were stained with TMRE (200 nM; 30 min), treated with CBD (0–60 μM) or FCCP (10 μM) for 10 min, washed, resuspended in experimental buffer (KCl 125 mM, KH_2_PO_4_ 1 mM, TrisHCl 10 mM, glutamate 5 mM, malate 2.5 mM, EGTA 1 mM, CaCl_2_ 0.7 mM) and placed in a 96-well plate. Fluorescence was assayed using a GloMax Discover plate reader, by exciting the sample at 549 nm and collecting the emission at 575 nm. Data from independent experiments were averaged and the effect of CBD or FCCP on membrane potential value is defined as % of TMRE fluorescence with respect to control.

### Detection of autophagic flux by LC3 immunoblotting

During autophagy, the amount of LC3-I decreases and that of LC3-II increases. But at the late phase after autophagolysosome formation LC3-II disappears being degraded by lysosomal proteases. If cells are treated with lysosomal protease inhibitors or with drugs inhibiting autophagosome-lysosome fusion, LC3-II degradation is prevented. Thus, LC3-II amount at a certain time point does *per se* serve a measure of the total autophagic flux. This flux should be more accurately evaluated by comparison of the amount of LC3-II between samples in the presence and absence of lysosomal protease inhibitors or compounds preventing autophagosome-lysosome fusion^15,44^. CQ was shown to prevent autophagosome-lysosome fusion^16^ and was used therefore in the present work.

For Western blot analysis, cells after corresponding treatments (CQ, CBD, or CQ and CBD combination) were harvested and lysed with RIPA buffer (Tris-HCl 25 mM, pH 7.6, NaCl 150 mM, EDTA 5 mM, NP-40 1%, sodium deoxycholate 1%, SDS 0.1%), supplemented with protease inhibitors (11697498001, Complete, Roche). For protein quantification, BCA Protein Assay Kit (Sigma) was used. For each sample, 15 µg/line of protein were loaded on a 15% SDS-PAGE gel. After electrophoresis (100 V, ~2 h), proteins were transferred onto PVDF membranes. Membranes were blocked for 1 hour with 5% BSA in TBS Tween-20 buffer (TBS-T) and incubated overnight at 4 °C with anti-human LC3 rabbit antibodies (Novus-Biologicals, NB100-2220, dilution 1:3000) and mouse monoclonal anti-human GAPDH antibodies (SCBT, sc-47724, dilution 1:1000) as a loading control. As secondary antibodies, HRP-conjugated goat anti-rabbit IgG (Novus-Biologicals, NBP2-30348H, dilution 1:3000) and HRP-conjugated anti-mouse IgGκ (SCBT, sc-516102, dilution 1:1000) were used for LC3 and GAPDH, respectively. Membranes were incubated with secondary antibodies over 1 h at room temperature, followed by incubation with the ECL detection reagent (Bio-Rad, 170-5061). Protein bands were visualized with Bio-Rad Universal Hood II system and analyzed with Image Lab 5.0 software.

### Autophagic flux measurement with mCherry-GFP-LC3

To measure autophagic flux at the single cell level, Jurkat cells, stably expressing tandem mCherry-GFP-LC3, were used^45^. Cells were cultured in the presence of CBD, CQ or their combination for 2, 4, and 24 h. After these periods, cells were collected by centrifugation (100 × *g*), suspended in PBS and placed in a in home-made coverslips-bottomed chambers for microscopy imaging. Double positive mCherry + /GFP + puncta represented autophagosomes, whereas fusion with the lysosome (autophagolysosomes) caused quenching of the pH-sensitive GFP, resulting in appearance of mCherry + GFP- puncta. CQ prevents GFP quenching by inhibiting autophagosome-lysosome fusion^16^. Samples were analyzed by confocal microscopy (LSM700, Carl Zeiss). Alternatively, custom-made confocal microscope (Solamere Technology Group, Salt Lake City, USA) based on a Yakogawa spindisk confocal scan head (CSUX1M1, Yokogawa Electronic Co., Tokio, Japan), equipped with solid state Coherent Obis lasers (405, 488, 561 and 640 nm) was used. Autophagy flux was evaluated by counting red (mCherry+) and green (GFP+) puncta (0.5–3 µm^2^) in the cells. Data analysis was performed using “Particle analysis” tool of ImageJ software, as reported by others^46–48^. Three fields for each condition (10–20 cells/ field) in at least three independent experiments were analyzed. Data are presented as mean of LC3 puncta/ cell or as mean of mCherry/GFP ratio.

### Protein-ligand docking

To explore binding sites for CBD within human VDAC1 channel, molecular docking analysis was performed, using Molegro virtual docker 6.0 software. Chemical structure of CBD was obtained from Pubchem (NIH, 644019), whereas hVDAC1 channel structure was downloaded from the protein data bank (PDB, 2JK4). For the beginning, three main cavities for hVDAC1 were defined and explored as possible CBD-interacting sites. MolDock Optimizer was selected as a search algorithm, 20 runs (number of times that the docking is repeated for each ligand) was chosen, and 1000 ligand binding conformations were established. From them, the best 5, based on their docking score (MolDock Score), were selected for each cavity. For all 15 selected binding conformations the contribution of each participating residue in the CBD binding was evaluated (Ligand map > Ligand Energy Inspector). Finally, basing on Total energy/MolDock Score, the best binding conformation was revealed and plotted by creating backbone visualization in the workspace.

## Supplementary information

Supplementary movie 1

Supplementary Table 1

Supplementary Figure 1
