# Supplementary material for: Cannabidiol directly targets mitochondria and disturbs calcium homeostasis in acute lymphoblastic leukemia
Source: Cell Death Dis. 2019 Oct 14;10(10):779. doi: 10.1038/s41419-019-2024-0 (PMC6791884; doi:10.1038/s41419-019-2024-0)
Supplement: Supplementary file 2 — Supplementary Table 1 [file 41419_2019_2024_MOESM2_ESM.docx]

| **Ligand pose CBD:644019** | **Cavity** | ***E*_total_ (MolDock Score)** | **Main hVDAC-1 interacting residues** |
| --- | --- | --- | --- |
| [00]644019 | 1 | -94.6131 | *Thr 119, Asp 131  ^#^Thr 9, Asp 103, Thr 119, Asn 127, Gly 129, Asp 131 |
| [01]644019 | 1 | -95.3599 | *Arg 18  ^#^Arg 18, Phe 21, Thr 22, Thr 251 |
| [02]644019 | 1 | -94.7971 | *Thr 9,  ^#^Thr 9, Lys 122, Asn 127, Asp 131 |
| [03]644019 | 1 | -914805 | *Thr 119 ^#^Thr 119, Gly 120, Asn 127, Asp 131 |
| [04]644019 | 1 | -93.5936 | *Thr 9, Tyr 10, Asp 131 ^#^Thr 9, Tyr 10, Asn 127, Asp 131, Ala 144 |
| [00]644019 | 2 | -104.968 | ^#^Asn 210, Phe 222, Gly 223, Asn 241 |
| [01]644019 | 2 | -102.664 | *Gly 195  ^#^Asn 186, Gly 195, Ser 196, Leu 211, Arg 221, Phe 222 |
| [02]644019 | 2 | -105.828 | *Gly 195  ^#^Asn 186, Ser 196, Leu 211, Phe 222 |
| [03]644019 | 2 | -106.757 | *Gly 195, Ser 196, Leu 211  ^#^Asn 188, Glu 192,Gly 194, Gly 195, Ser 196, Asn 210, Leu 211, Ala 212 |
| [04]644019 | 2 | -103.628 | *Gly 195  ^#^Asn 186, Gly 195, Ser 196, Arg 221 |
| **[00]644019** | **3** | **-159.797** | ***Gly 175 ^#^Thr 9, Asp 12, Leu 13, Val 146, Leu 153, Gln 157, Gly 175, Gln 182, His 184** |
| [01]644019 | 3 | -151.815 | *Thr 9, Gly 175 ^#^Pro 8, Thr 9, Asp 12, Leu 13, Val 146, Leu 153, Gly 155, Gln 157, Gly 175, Gln 182, His 184 |
| [02]644019 | 3 | -144.818 | *Pro 8, Gly 175 ^#^Thr 9, Leu 153, Gln 157,Ala 173, Val 174, Gly 175, Asn 186 |
| [03]644019 | 3 | -149.071 | *Thr 9, Gly 175 ^#^Thr 9, Asp 12, Leu 13, Lys 15, Val 146, Leu 153, Gln 157, Gly 175, Gln 182, His 184 |
| [04]644019 | 3 | -139.347 | *Pro 8, Gly 175 ^#^Thr 9, Ala 11, Asp 12, Leu 153, Gly 175, Leu 183, His 184 |

Supplementary Table 1. CBD-hVDAC-1 docking

*Hydrogen bond interaction ^#^steric interaction
