# Supplementary figures and images for: Cannabidiol directly targets mitochondria and disturbs calcium homeostasis in acute lymphoblastic leukemia
Source: Cell Death Dis. 2019 Oct 14;10(10):779. doi: 10.1038/s41419-019-2024-0 (PMC6791884; doi:10.1038/s41419-019-2024-0)

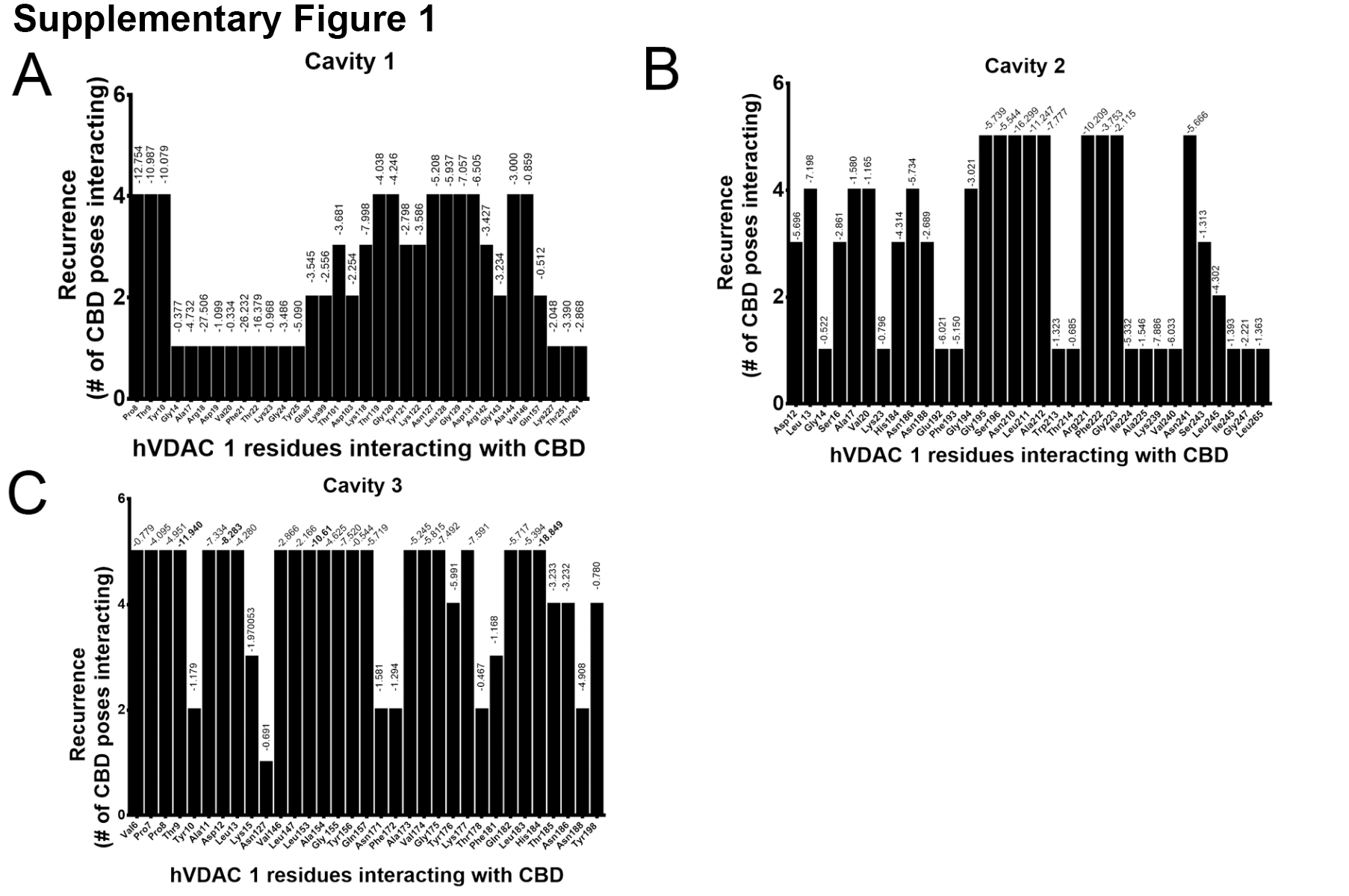

Supplement: Supplementary file 3 — Supplementary Figure 1 [file 41419_2019_2024_MOESM3_ESM.jpg]
